# Advances in Molecular Mechanisms for Traditional Chinese Medicine Actions in Regulating Tumor Immune Responses

**DOI:** 10.3389/fphar.2020.01009

**Published:** 2020-07-08

**Authors:** Han Huang, Jiansong Fang, Xiude Fan, Tatsunori Miyata, Xiaoyue Hu, Lihe Zhang, Liangren Zhang, Yimin Cui, Zhenming Liu, Xiaoqin Wu

**Affiliations:** ^1^ State Key Laboratory of Natural and Biomimetic Drugs, School of Pharmaceutical Sciences, Peking University, Beijing, China; ^2^ Science and Technology Innovation Center, Guangzhou University of Chinese Medicine, Guangzhou, China; ^3^ Center for Liver Disease Research, Department of Inflammation and Immunity, Cleveland Clinic, Cleveland, OH, United States; ^4^ Department of Pharmacy, Peking University First Hospital, Beijing, China

**Keywords:** Traditional Chinese medicine (TCM), cancer cells, tumor immunosuppressive microenvironment (TIM), immune response, synergistic effect

## Abstract

Traditional Chinese medicine (TCM) has been developed for thousands of years with its various biological activities. The interest in TCM in tumor prevention and treatment is rising with its synergistic effect on tumor cells and tumor immunosuppressive microenvironment (TIM). Characteristic of TCM fits well within the whole system and multi-target cancer treatment. Herein we discuss the underlying mechanisms of TCM actions in TIM *via* regulating immunosuppressive cells, including restoring the antigen presentation function of dendritic cells, enhancing NK cells-mediated killing activity, restraining the functions of myeloid cell-derived suppressor cells, and inhibiting cancer-associated fibroblasts. TCM also regulates tumor progression through enhancing immune response, preventing immune escape and inducing cell death of tumor cells, which triggers immune response in nearby cells. In addition, we discuss TCM in clinical applications and the advantages and disadvantages of TCM in cancer prevention and treatment, as well as current therapeutic challenges and strategies. It might be helpful for understanding the therapeutic potential of TCM for cancer in clinic.

## Introduction

Tumor microenvironment (TME) plays a crucial role in the development and migration of tumor. Accumulating evidence show that cancer growth and metastasis occur as a result of disruption between cancer cells and the TME (Fidler, 2003). Immune cells in TME promote tumor progression developing a tumor immunosuppressive microenvironment (TIM), which releases immunosuppressive factors that alter the phenotype and function of immune cells. Some high risk factors in TME, including inflammatory cytokines, hyperosmosis, acid environment, and hypoxia promote TIM formation. Targeted organs can release various cytokines to induce angiogenesis, lymphangiogenesis and cell proliferation, resulting in metastasis formation. Meanwhile, tumor also regulates TME for their own survival. Therefore, we cannot simply consider one target or one pathway in tumor therapeutics. Tumor cells and TME should be taken into account as a whole system. Multi-target strategy has the potential to solve this existing problem.

Traditional Chinese medicine (TCM) is one of multi-target strategies for cancer therapeutics (Li and Lin, 2011) based on its overall adjustment treatment. It could suppress tumor growth and recurrence by inhibiting proliferation of cancer cells *per se*, and fine-tuning the homeostasis of TIM. Many Chinese herbs can reduce the toxicity of chemotherapy and radiotherapy, ultimately prolonging the overall survival time (Li et al., 2012). A number of studies elucidate the underlying mechanisms for TCM synergistic influence in mediating cancer cells as well as TME. Therefore, we here summarize advances in molecular mechanisms of TCM action on tumor, especially on regulating tumor immunity.

## TCM Could Regulate TIM

TME is a complex system with multiple components, such as nontumor cells and extracellular matrix. Nontumor cells mainly consist of innate immune cells including macrophages, natural killer (NK) cells, dendritic cells (DCs), etc., acquired immune cells (T and B cells), myeloid-derived suppressor cells (MDSCs), fibroblasts (Egeblad et al., 2010). TME assists tumor cells to escape immune surveillance, and combines with extracellular matrix proteins and matrix-degrading enzymes to form TIM (Sun, 2015). TCM could reverse the inhibitory phenotype of immune cells, restore the function of innate immune cells (**Table 1**) and adaptive immune cells (**Table 2**) in the TIM.

**Table 1:** Effect of TCM on crosstalk between tumor cells and innate immune cells.

TCM	Tumor type	Effects on tumor cells	Main immune mechanisms	Ref.
**DCs**				
SL formula	*In vivo*, a murine xenograft model of B16F10 melanoma	Could suppress tumor growth in melanoma- bearing mice	Inhibiting the activation of STAT3 and STAT3-targeted immunosuppressive cytokines; increasing recruitment of DCs to melanoma tissues and spleens to enhance immune response.	(Liu et al., 2019)
Am and/or Cp (TCM components)	*In vivo*, a murine orthotopic mammary carcinoma resection model	Could enhance efficiency of DC-based vaccine against metastasis of 4T1 mammary carcinoma and the improved survival in mice	Increasing the expression of CD40, CD80 and CD86 in DCs and CD4^+^ and CD8^+^ T-cell proliferation	(Chang et al., 2015)
**NK cells**				
YPF formula	*In vivo*, a LLC-xenografted murine model	Could inhibit the growth of LLC and prolong the survival of tumor-bearing mice	Downregulating the protein levels of indoleamine 2,3-dioxygenase, TGF-β, and IL-10, which promoted tumor infiltration and killing capability of NK cells to LLC	(Luo et al., 2016)
Lupeol (TCM monomer)	*In vitro*, gastric cancer cell lines BGC823, N87 and HGC27	Could inhibit the proliferation of gastric cancer cells	Inducing the proliferation and promoting the killing power of NK cells through the upregulation of PFP, IFN-γ and CD107a in NK cells	(Wu et al., 2013)
PSG-1 (TCM monomer)	*In vitro* and *in vivo*, S-180 cells and a murine xenograft model of sarcoma	Could inhibit S-180 cells *in vitro* and decrease sarcoma tumor weight and induce apoptosis in mice	Increasing production of cytokines in Th1 cells and enhancing the cytotoxic activity of NK and CTL cells in mice by TLR4	(Yu et al., 2015)
**Macrophages**				
BFD formula	*In vitro* and *in vivo*, A549 and H1975 cells and murine xenograft models of NSCLC	Could inhibit tumor growth and prolong the survival in mice	Blocking the crosstalk between TAMs and cancer cells through decreasing IL-10 and PD-L1	(Pang et al., 2017)
Baicalin (TCM monomer)	*In vivo*, a murine orthotopic HCC implantation model	Could inhibit tumor growth of HCC	Inducing the repolarization of TAM to M1-like phenotype and promoting the production of pro-inflammatory cytokines in tumor; induction of autophagy and activation of RelB/p52	(Tan et al., 2015)
**MDSCs**				
SGJP formula	Patients with breast cancer, a murine model of 4T1 mammary cancer	MDSCs in patients with breast cancer were positively associated with cancer progression; Anti-tumor activity in mice	Had an inhibitory effect on Gr-1^+^ CD11b^+^ myeloid immunosuppressor cells; preventing MDSCs-induced IL-4, IL-13 and TGF-β expression and apoptosis of CD8^+^ T cells, as well as enhancing inflammatory responses of NKT cells by JAK-STAT signaling	(Guo et al., 2015)

SL, Styphnolobium japonicum (L.) Schott [Fabaceae] and Lonicera japonica Thunb [Caprifoliaceae]); Am, a mixed polysaccharide fractions from the root of Astragalus mongholicus Bunge [Fabaceae]; YPF, Yu-Ping-Feng; LLC, Lewis lung cancer; PSG-1, Ganoderma atrum polysaccharide; BFD, Bu Fei Decoction; NSCLC, non-small cell lung cancer; HCC, hepatocellular carcinoma; SGJP, Shugan Jianpi.

**Table 2:** Effect of TCM on crosstalk between tumor cells and adaptive immune cells.

TCM	Tumor type	Effects on tumor cells	Main mechanisms	Ref.
**Tregs**				
SYY formula	*In vivo*, a murine xenograft model of liver cancer	Could inhibit growth and lung metastasis of liver cancer	Reducing the proportion of Treg and TGF-β1 expression in spleen, peripheral blood and tumor tissue	(Zhang Q. B. et al., 2016)
GP (TCM component)	*In vivo*, a murine xenograft model of hepatocarcinoma H22	Could inhibit tumor growth of hepatocarcinoma	Reducing the frequency of CD4^+^CD25^+^ Tregs and Foxp3 expression, but increasing the ratio of Th1/2 cytokines in serum	(He et al., 2011)
**B cells**				
Matrine (TCM monomer)	*In vitro*, human ALL B-lymphocytes	Could induce human ALL B-lymphocytes apoptosis	Upregulating the proapoptotic protein Bax while downregulating the anti-apoptotic protein Bcl-2 in human ALL B-lymphocytes	(Aghvami et al., 2018)

SYY, Songyou Yin); GP, Polysaccharide from Glycyrrhiza uralensis Fisch. ex DC. [Fabaceae]; ALL, acute lymphoblastic leukemia.

### TCM Could Regulate Innate Immune Response

Since TCM could increase expression of inflammatory factors and decreases production of immunosuppressive cytokines, DCs can proliferate and differentiate normally, the ability of antigen presentation can be restored; the number of activated NK cells is increased; the number of M2 macrophages is decreased, and its ability of SLE (Yu et al., 2015; Li et al., 2017; Pang et al., 2017).

DCs are the main antigen-presenting cells (APCs) in innate and adaptive immunity; they present antigens to T cells to initiate the immune cycle at baseline (Chen and Mellman, 2013). DCs endocytose cell debris or dead tumor cells and then present tumor antigens to lymph nodes to activate T cells. However, in TIM, DCs are unable to proliferate and differentiate, leading to inhibit antigen presentation and activation of T cells (Gardner and Ruffell, 2016). Defective DCs have been found in various cancers, such as pancreatic cancer, non-small cell lung cancer (NSCLC), and hepatocellular carcinoma (HCC) and cervical squamous intraepithelial lesions (Gabrilovich, 2004; Bellone et al., 2006; Lee et al., 2006; Ormandy et al., 2006; Perrot et al., 2007). There is a TCM formula (SL), comprising *Styphnolobium japonicum* (L.) Schott [Fabaceae] and *Lonicera japonica* Thunb [Caprifoliaceae], which is traditionally used for melanoma treatment (Li et al., 2017). Using a mouse xenograft model of B16F10 melanoma, they found that an ethanolic extract of SL (SLE) could dramatically suppress tumor growth in melanoma-bearing mice, partially by inhibiting the activation of STAT3 and STAT3-targeted immunosuppressive cytokines, which involved in tumor growth and immune evasion. These anti-melanoma effects of SLE were also associated with increased recruitment of DCs to B16F10 melanoma tissues and mouse spleens to enhance tumor immune response (Liu et al., 2019). DC-based vaccines are novel emerging strategy for cancer immunotherapies. Treatment of Am, Cp or [Am + Cp] (two polysaccharide fractions from the root of *Astragalus mongholicus* Bunge [Fabaceae] and *Codonopsis pilosula* (Franch.) Nannf. [Campanulaceae]) could increase the expression of CD40, CD80 and CD86 in DCs and CD4^+^ and CD8^+^ T-cell proliferation, resulting in the enhanced efficiency of DC-based vaccine against metastasis of 4T1 mammary carcinoma and the improved survival in mice (Chang et al., 2015).

In addition to DCs, NK cells are also dysfunctional in TIM (Cekic et al., 2014). It has been reported that a typical Chinese herbal decoction Yu-Ping-Feng (YPF, contains *A. mongholicus* Bunge [Fabaceae]), *Atractylodes macrocephala* Koidz. [Asteraceae]), *Saposhnikovia divaricata* (Turcz. ex Ledeb.) Schischk. [Apiaceae]) could downregulate the protein levels of indoleamine 2,3-dioxygenase, TGF-*β*, and IL-10, which promoted tumor infiltration and killing capability of NK cells to LLC (Lewis lung cancer) in mice. Meanwhile, YPF also could inhibit the growth of LLC and prolong the survival of tumor-bearing mice (Luo et al., 2016). Lupeol is a natural secondary metabolite isolated and purified from *Tamarindus indica* L. [Fabaceae] (Saleem, 2009). Lupeol could inhibit the proliferation of gastric cancer cell lines BGC823, N87 and HGC27 by inducing the proliferation of NK cells and promoting the killing power of NK cells *in vitro*. Further study showed Lupeol could upregulate the expression of PFP, IFN-γ and CD107a in NK cells (Wu et al., 2013). *Ganoderma atrum* has been used for thousands of years as a traditional medicine. A polysaccharide is extracted from *G. atrum*, named as PSG-1. PSG-1 could inhibit S-180 cells *in vitro*, and decrease sarcoma tumor weight and induce apoptosis in mice. By using C3H/HeN (WT) and C3H/HeJ (TLR4-deficient) mice, they further found that PSG-1 could increase production of cytokines in Th1 cells, and enhance the cytotoxic activity of NK and CTL cells in WT, but not TLR4-deficient mice, suggesting that PSG-1-mediated antitumor activity is likely dependent on TLR4 (Yu et al., 2015).

Macrophages exhibit different phenotypes upon different environmental stimuli, mainly including classically activated macrophages (M1) as well as alternatively activated macrophages (M2). While studies show that Tumor-associated macrophages (TAMs) are able to exhibit either phenotype, researchers tend to consider TAMs as M2-like macrophages. TAMs contribute to TIM through producing cytokines, proteases, chemokines and growth factors, as well as inducing the release of inhibitory immune checkpoint proteins in T cells (Siamon et al., 2015). A classical TCM formula Bu Fei Decoction (BFD) comprising *Morus alba* L. [Moraceae], *Aster tataricus* L.f. [Asteraceae], *A. mongholicus* Bunge [Fabaceae], *Rehmannia glutinosa* (Gaertn.) DC. [Orobanchaceae], *Schisandra chinensis* (Turcz.) Baill. [Schisandraceae], and *C. pilosula* (Franch.) Nannf. [Campanulaceae] alleviated lung cancer-related symptoms in clinic. Another study in murine xenograft models of non-small cell lung cancer (NSCLC) using A549 and H1975 cells showed BFD could inhibit tumor growth and prolong the survival in mice by blocking the crosstalk between TAMs and cancer cells through decreasing IL-10 and PD-L1 *in vitro* and *in vivo* (Pang et al., 2017). In addition, baicalin, a major bioactive compound extracted from *Scutellaria baicalensis* Georgi [Lamiaceae] could inhibit tumor growth in a mouse orthotopic model of HCC by inducing the repolarization of TAM to M1-like phenotype and promoting the production of pro-inflammatory cytokines in tumor. These effects were associated with the induction of autophagy and activation of RelB/p52 (Tan et al., 2015).

MDSCs, one of main components in the TIM, are generated in the bone marrow, and migrated to peripheral lymphoid organs and tumor tissues and differentiate to DCs or macrophages (Jia Y. et al., 2012). Recent studies showed that MDSCs in patients with breast cancer were positively associated with cancer progression (Guo et al., 2015). Shugan Jianpi (SGJP) formula consists of *Paeonia lactiflora* Pall. [Paeoniaceae], *Stauntonia angustifolia* (Wall.) R.Br. ex Wall. [Lardizabalaceae], *Bupleurum chinense* DC. [Apiaceae], *Curcuma aromatica* Salisb. [Zingiberaceae], *A. mongholicus* Bunge [Fabaceae], *Prunella vulgaris* L. [Lamiaceae], *Glycyrrhiza uralensis* Fisch. ex DC. [Fabaceae] and *Panax notoginseng* (Burkill) F.H.Chen [Araliaceae]. SGJP has an inhibitory effect on Gr-1^+^ CD11b^+^ myeloid immunosuppressor cells in a murine model of 4T1 mammary cancer. Furthermore, SGJP could prevent MDSCs-induced IL-4, IL-13 and TGF-β expression and apoptosis of CD8^+^ T cells, and enhance inflammatory responses of NKT cells by JAK-STAT signaling (Guo et al., 2015).

### TCM Could Regulate Adaptive Immune Response

In addition to innate immune cells, adaptive immune cells are also reversed by TCM. Regulatory T cells (Tregs) now have gotten more and more attention as their impact on inhibiting tumor-associated immune responses. Therefore, strategies targeting Tregs to improve adaptive immune response may be promising for cancer treatment (Chaudhary and Elkord, 2016). Songyou Yin (SYY), a known herbal TCM formula, contains *Salvia miltiorrhiza* Bunge [Lamiaceae]), *A. mongholicus* Bunge [Fabaceae], *Lycium barbarum* L. [Solanaceae], *Crataegus pinnatifida* Bunge [Rosaceae], and *Trionyx sinensis* Wiegmann. SYY has a therapeutic potential in multiple cancers (Liu et al., 2012; Wang et al., 2015), including HCC (Xiong et al., 2010; Jia Q. A. et al., 2012; Jia Q. A. et al., 2013). In addition, SYY also could inhibit tumor growth through Treg immunomodulation. In a murine xenograft model of liver cancer (Hepa1-6), SYY with moderate swimming (MS) could inhibit growth and lung metastasis of liver cancer and prolong mouse survival. Further study found that SYY and MS could increase the ratio of CD4^+^ to CD8^+^, but reduce the proportion of Treg and TGF-β1 expression in spleen, peripheral blood as well as tumor tissue (Zhang Q. B. et al., 2016). In a murine xenograft model of hepatocarcinoma H22, administration of Polysaccharide from *G. uralensis* Fisch. ex DC. [Fabaceae] (GP) could reduce the frequency of CD4^+^CD25^+^ Tregs and Foxp3 expression, but increase the ratio of Th1/2 cytokines in serum, which partially contributes to GP-mediated inhibition of tumor growth (He et al., 2011).

B cells are critical in humoral immunity. Until recently, some studies have found B cells infiltration in solid tumors. However, the role of B cells in solid tumors is conflicting (Flynn et al., 2017; Kaplon and Dieu-Nosjean, 2018). Little is known about TCM in regarding B cell-mediated immune responses. Matrine is a medicinal herb derived from *Sophora flavescens* Aiton [Fabaceae]. One study identified the underlying mechanism for Matrine’s anti-cancer effects in human acute lymphoblastic leukemia (ALL). Treatment of B cells from human ALL with Matrine could induce ROS generation and mitochondrial swelling, and cause a decline in mitochondrial membrane potential, thereby inducing apoptosis by upregulation of the proapoptotic protein Bax and downregulation of the anti-apoptotic protein Bcl-2 (Aghvami et al., 2018).

## TCM Could Inhibit Cancer-Associated Fibroblasts

Under physiological conditions, fibroblasts are able to secrete multiple cytokines, and their plasticity is high, which is essential for maintaining cell homeostasis and repairing tissue damage (Jacob et al., 2012). However, cells with similar morphology to myofibroblasts were found in the cell matrix of solid tumors, which are referred to as cancer-associated fibroblasts (CAFs) (Tremblay, 1979). CAFs can promote tumor invasion and migration by changing the phenotype of cancer cells. CAFs also promote tumor angiogenesis and affect the TME to facilitate cancer progression (Santi et al., 2018). Additionally, accumulating evidence have shown that CAFs mediate epithelial-to-mesenchymal transition (EMT) in tumor cells (Christofori, 2006; Chen and Song, 2019). Therefore, strategies inhibiting CAFs are one of important approaches to suppress tumor progression (**Table 3**).

**Table 3:** Inhibition of TCM on CAFs.

TCM	Tumor type	Effects on tumor cells	Main mechanisms	Ref.
ARS and DHA (TCM monomer)	*In vitro* and *in vivo*, L-929-CAFs and a murine orthotopic breast cancer implantation model	Could reverse breast cancer cell-CAFs from activated to inactivated state	Suppressing the TGF-β signaling to inhibit the interaction between tumor and TME.	(Yao et al., 2018)
Curcumin (TCM monomer)	*In vivo*, a nude mouse xenograft model of pancreatic cancer	Could inhibit EMT and metastasis of pancreatic cancer cells	Inhibiting CAFs	(Wang et al., 2017; Qu et al., 2018)
PP-1 (TCM component)	*In vitro*, prostate-CAFs	Could inhibit the growth of prostate-CAFs	Induction of autophagy by increasing the activation of Beclin-1 and LC3	(Han S.Y. et al., 2016)

ART, artesunate; DHA, dihydroartemisinin; CAFs, cancer-associated fibroblasts; PP-1, Polysaccharide extracted from Polygonatum odoratum (Mill.) Druce [Asparagaceae].

Artemisinin (ART) is a chemical extract from Chinese herb *Artemisia annua* L. [Asteraceae], which has potent anti-malarial and anti-cancer activity. ART derivatives artesunate (ARS) and dihydroartemisinin (DHA) could reverse L-929 breast cancer cell-CAFs from activated to inactivated state through inhibition of TGF-β signaling, which resulted in a disruption of the interaction between tumor and TME. *In vivo* data also showed that ART derivatives also could suppress CAFs-induced growth and metastasis of breast cancer in an orthotopic model (Yao et al., 2018). Curcumin is a chemical component extracted from *Curcuma longa* L. [Zingiberaceae]. Studies have showed that curcumin has blood lipid lowering, antitumor, anti-inflammatory, choleretic and anti-oxidant effects (Lestari and Indrayanto, 2014). Curcumin could inhibit EMT and metastasis of pancreatic cancer cells by inhibiting CAFs in a nude mouse model of pancreatic cancer (Wang et al., 2017; Qu et al., 2018). Polysaccharide (PP-1) are extracted from *Polygonatum odoratum* (Mill.) Druce [Asparagaceae], a perennial herb of the lily family. PP-1 could selectively inhibit the growth of prostate-CAFs without affecting normal broblasts through induction of autophagy by increasing Beclin-1 and LC3, key autophagy-related proteins (Han S. Y. et al., 2016).

## TCM Could Enhance Immune Response Towards Tumor Cells

Immune response of tumor cells is critical for tumor progression. Furthermore, cell death can trigger immune responses of nearby cells through releasing damage-associated molecular patterns (DAMPs), including cytokines or chemokines (Lau et al., 2020). Accumulating evidence shows that TCM could enhance immune responses and protect from immune escape (**Table 4**), and induce cell death of tumor cells (**Table 5**).

**Table 4:** Effect of TCM on immune response of tumor cells.

TCM	Tumor type	Effects	Main mechanisms	Ref.
**Upregulation of Classic MHC Molecules**				
EPS (TCM component)	*In vitro*, DCS cells	Could improve the expression of MHC-II, CD40, CD80, and CD86 in DCS cells and their ability of antigen uptake as well as secretion of IL-12 and TNF-α	Inhibiting phosphorylation of JAK2 and STAT3 and promoting the NF-κB signal pathway	(Song et al., 2013)
ISD formula	*In vivo*, in spleen-deficient liver cancer rats	Could suppress the development of cachexia caused by transplantable tumor and improve the survival of mice	Increasing MHC I/II expression in liver tissues	(Wang et al., 2008; Li et al., 2014)
**Inhibition of PD-1/PD-L1 signaling**				
BFD formula	*In vitro* and *in vivo*, A549 and H1975 cells and murine xenograft models of NSCLC	Could inhibit tumor growth and prolong the survival in mice	Blocking the crosstalk between TAMs and cancer cells through decreasing IL-10 and PD-L1	(Pang et al., 2017)
QYSL formula	*In vivo*, a LLC-xenografted murine model	Could inhibit tumor growth	High dose QYSL inhibited tumor growth by reducing PD-1 in spleen and PD-L1.	(Zhang X. et al., 2016)
GQD formula	*In vivo*, a murine xenograft model of CT26 CRC	Could inhibit tumor growth of CRC and modulate the gut microbiome composition.	Combination therapy with GQD and anti-PD-1 induced the frequency of CD8+ T cells in tumor tissues and peripheral blood. They also increased IFN-γ and IL-2, but decreased PD-1.	(Lv et al., 2019)
**Inhibition of CSCs**					
PZH formula	*In vitro*, HT-29 CRC stem-like SP cells	Could reduce the population and viability and sphere-forming capacity of HT-29 SP cells	Inhibiting ABCB1 and ABCG2	(Wei et al., 2014)	
Huaier (TCM component)	*In vitro*, MCF7 breast cancer cells	Could decrease the viabilities, numbers, sizes of mammospheres and the proportion of cells expressing CD44^+^/CD24^-,^ and reduce the levels of stem cell markers	Partially dependent on the hedgehog pathway	(Wang et al., 2014)	
	*In vitro*, primary CRC cells (T1 and T2 cells)	Could inhibit the potential of spheroid formation and the population of ALDH−positive cell	Downregulating the Wnt/β−catenin pathway	(Zhang et al., 2013)	

EPS, the exopolysaccharide from an anamorph of Cordyceps sinensis; ISD, Invigorating Spleen and Detoxification Decoction; DCS, dendritic cell sarcoma; GQD, Gegen Qinlian decoction; CRC, colorectal cancer; BFD, Bu Fei Decoction; NSCLC, non-small cell lung cancer; QYSL, Qiyusanlong decoction; LLC, Lewis lung cancer; CSCs, cancer stem cells; PZH, Pien Tze Huang; Huaier, Trametes robiniophila Murr.; ALDH, aldehyde dehydrogenase.

**Table 5:** Effect of TCM on cell death of tumor cells.

TCM	Tumor type	Effects	Main mechanisms	Ref.
**Induction of apoptosis**				
AST (TCM component)	*In vitro*, HT-29 CRC cells	Could induce the extrinsic apoptotic cascade and caused cell cycle arrest	Regulating both mTOR and ERK signaling pathways, inhibition of NF-kappaB is a critical latter event	(Auyeung et al., 2010)
LJGP (TCM component)	*In vitro*, AGS gastric cancer cells	Could inhibit tumor growth and induce apoptosis by upregulating pro-apoptotic Bax, and downregulating anti-apoptotic Bcl-2 and IAP family members, as well as activation of caspase-3/9	downregulating telomerase activity and prostaglandin E2 synthesis by decreasing COX-2	(Ho et al., 2011)
PHY906 formula	*In vivo*, a nude mouse xenograft model of HepG2	Could increase cell apoptosis	Increasing mouse FasL and human FasR expression	(Lam et al., 2015)
YWKLF formula	*In vitro*, human gastric cancer MGC-803 cells	The sera from rabbits orally administered with YWKLF induced cell apoptosis	Inducing mitochondrial dysfunction, increasing the expression of Fas and Bax, and reducing the mRNA levels of FasL	(Li et al., 2008)
**Induction of autophagy**				
Bufalin (TCM monomer)	*In vitro*, human hepatoma cancer Huh7, Hep3B and HA22T cells	Could inhibit the proliferation, regulate the cell death program, and induce autophagy	Increasing TNF, MAPK and BECN-1 and ATG8, and decreasing Bcl-2 and Bid	(Hsu et al., 2013)
DHA-37 (TCM monomer)	*In vitro* and *in vivo*, multiple human cancer cell lines including A549 SGC-7901, and a murine xenograft model of A549	Could trigger ACD in A549 cells and inhibit tumor growth *in vivo*	Activating the MAPK signal and upregulating HMGB1 *in vitro* and increasing p-ERK, p-P38, HMGB1, and LC3 in tumor tissue	(Liu et al., 2018)
PGB (TCM component)	*In vitro*, human lung carcinoma A549 cells	Could induce ACD of A549 cells	Suppressing the AKT/mTOR pathway, and activating the AMPK and MAPK pathways	(Ma et al., 2016)
FOJ and SSOJ (Components of OJ formula)	*In vitro*, human lung carcinoma A549 cells	Could induce autophagy of A549 cells by upregulating protein levels of LC3-II and mRNA levels of Atg-3, Atg-7, Beclin-1 and LC3-II/I	Inhibiting the PI3K/Akt/mTOR signaling	(Chen J. et al., 2017)
**Induction of necroptosis**				
Resibufogenin (TCM monomer)	*In vivo*, a murine xenograft model of CRC	Could suppress tumor growth and metastasis	Induction of necroptosis through increasing RIP3 and pMLKL	(Han et al., 2018)
Shikonin (TCM monomer)	*In vitro*, rat C6 and human U87 glioma cells	Could induce necroptosis	Mediated by oxidative stress and RIP1 signaling	(Huang C. et al., 2013)
**Induction of multiple cell death pathways**				
SB (TCM component)	*In vitro* and *in vivo*, human lung cancer CL1-5 cells and a murine xenograft model of lung cancer	Could suppress proliferation and angiogenesis, and increase apoptosis and autophagy	ER stress-, intrinsic mitochondrial-, P38/SIRT1-regulated cell apoptosis through G2/M phase arrest and extrinsic Fas/FasL-mediated pathways	(Chen C.C. et al., 2017)
HLP (TCM component)	*In vitro*, human malignant melanoma A375 cells	Could induce apoptosis and ACD in A375 cells	Increasing the caspases cleavages, Bcl-2, and Fas/FasL activation and ATG5, Beclin1, and LC3-II	(Chiu et al., 2015)
Shikonin (TCM monomer)	*In vitro* and *in vivo*, mouse stage IV mammary carcinoma 4T1-luc2 cells and a murine model of mammary carcinoma	Could trigger RIP1- and RIP3-dependent necroptosis and autophagy, and stimulate the derived vaccine efficacy	Enhancing the surface DMAP activity and DC activation	(Lin et al., 2018)

AST, The total saponins of Astragalus mongholicus Bunge [Fabaceae]; CRC, colorectal cancer; LJGP, Glycoprotein isolated from Laminaria japonica; YWKLF, a herbal medicine formula Yang Wei Kang Liu; DHA-37, Dihydroartemisinin; ACD, autophagic cell death; HMGB1, high mobility group protein; PGB, the platycoside-containing butanol fraction of PG; FOJ, Flavonoids; SSOJ, steroidal saponins; OJ, Ophiopogon japonicus (Thunb.) Ker Gawl. [Asparagaceae]; SB, Scutellaria barbata D.Don [Lamiaceae]; HLP, Hibiscus leaf polyphenolic.

### TCM Could Prevent Tumor Cells From Immune Escape

#### TCM Could Increase Expression of Classic MHC Molecules

In general, MHC I presents tumor antigens to CD8^+^ T cells, also called cytotoxic T cells (CTLs), which kill tumor cells by cytolysis. In contrast, MHC II identifies tumor antigen peptides to CD4^+^ T helper cells, which trigger cell-mediated immunity. Classical MHC molecules (I and II) enhance the interplay between tumor cells and NK cells or CTLs by identifying the tumor antigen (Guo et al., 2015). However, malignant and immune cells in TME may down-regulate MHC I or II, but express nonclassical human leukocyte antigens (HLAs) including HLA-E/F/G that were associated with tumor cells escaping from T and NK cell-mediated recognition (Kochan et al., 2013). Exopolysaccharide (EPS) from an anamorph of *Cordyceps sinensis* could induce expression of MHC-II, CD40, CD80, and CD86 in dendritic cell sarcoma (DCS) cells, and enhance their ability of antigen uptake as well as secretion of IL-12 and TNF-α through decreasing p-JAK2 and p-STAT3 and increasing p-p65, suggesting that EPS may induce DCS cells to exhibit a mature phenotype, which is critical in initiating antitumor immunity (Song et al., 2013). Another TCM, Invigorating Spleen and Detoxification Decoction (ISD) (*Scutellaria barbata* D.Don [Lamiaceae], *G. uralensis* Fisch. ex DC. [Fabaceae], *C. longa* L. [Zingiberaceae], *B. chinense* DC. [Apiaceae], *A. macrocephala* Koidz. [Asteraceae], *Smilax glabra* Roxb. [Smilacaceae], and *C. pilosula* (Franch.) Nannf. [Campanulaceae]) could decrease the progression of cachexia and prolong the survival time in spleen-deficient liver cancer rat by increasing MHC I/II expression in liver tissues (Li et al., 2014).

#### TCM Could Inhibit PD-1/PD-L1 Signaling

Programmed cell death 1 (PD-1), as a currently well-known immune checkpoint, preferentially expressed on B and T cells, as well as other cells including DCs and monocytes (He et al., 2015). Under multiple physiological conditions, PD-1 modulates the activity of T cells in peripheral tissues by inducing inhibitory signals in immune system and maintains self-tolerance in inflammation or infection (Trivedi et al., 2015). However, PD-1/PD-L1 (ligand) pathway is utilized by the tumor cells to escape immunologic surveillance in the context of cancer (Bagley et al., 2015). Studies showed that PD-L1 was over-expressed in various tumor cells, including lung, ovarian and colon cancer (Keir et al., 2008). At present, there are accumulating TCM researches on PD-1/PD-L1. In addition to BFD that we mentioned above (Pang et al., 2017), another TCM formula, Qiyusanlong decoction (QYSL) (*A. mongholicus* Bunge [Fabaceae], *P. odoratum* (Mill.) Druce [Asparagaceae], *Solanum nigrum* L. [Solanaceae], *Scleromitrion diffusum* (Willd.) R.J.Wang [Rubiaceae], *Coix lacryma-jobi* L. [Poaceae], *Curcuma phaeocaulis* Valeton [Zingiberaceae], *Fritillaria cirrhosa* D.Don [Liliaceae], *Euphorbia helioscopia* L. [Euphorbiaceae], *Scolopendra*, *Pheretima*) also has effect on the PD-1/PD-L1 pathway in lung cancer. High concentration of QYSL could inhibit tumor growth by reducing PD-1 in spleen and PD-L1 in tumor in a murine xenograft model of LLC (Zhang X. et al., 2016). In a systemic pharmacological study, combination treatment of colorectal cancer (CRC) with anti-mouse PD-1 and GQD (a classical TCM formula Gegen Qinlian decoction, *Pueraria montana* var. lobata (Willd.) Maesen & S.M.Almeida ex Sanjappa & Predeep [Fabaceae], *S. baicalensis* Georgi [Lamiaceae], *Coptis chinensis* Franch. [Ranunculaceae]), *G. uralensis* Fisch. ex DC. [Fabaceae]) inhibited tumor growth in a murine xenograft model of CT26 CRC. Gut microbiota analysis revealed that GQD could modulate the gut microbiome composition. In particular, they found that combination therapy with GQD and anti-PD-1 induced the frequency of CD8^+^ T cells in tumor tissues as well as peripheral blood, and increased IFN-γ and IL-2, but decreased PD-1. These data indicate that the combination therapy effectively restores T-cell functions by suppressing inhibitory checkpoints (Lv et al., 2019).

#### TCM Could Inhibit CSCs

Cancer stem cells (CSCs), a small population of cells, are critical in tumor development and drug resistance, resulting in metastasis and cancer relapse. CSCs hardly express molecules that favor immune response, such as HLA-DR. This is the reason that CSCs becomes a major clinical challenge in cancer treatment (Zeuner et al., 2014). Therefore, strategy targeting or inhibiting CSCs is a novel and promising approach for cancer therapeutics (Guo et al., 2015).

Accumulating evidence indicates that TCM could reduce CSCs. For instance, PZH (*Abelmoschus moschatus* Medik. [Malvaceae], *Calculus bovis*, Snake Gall and *P. notoginseng* (Burkill) F.H.Chen [Araliaceae]), a well-known TCM formula has been prescribed for hundreds of years in China, could reduce the population of the HT-29 CRC stem-like SP cells in a dose-dependent manner, and reduce the viability and sphere-forming capacity of HT-29 SP cells. Mechanistically, PZH could inhibit ABCB1 and ABCG2 that are members of the ABC transporter superfamily contributing to the SP phenotype and multi-drug resistance (Wei et al., 2014). *Trametes robiniophila* Murr. (Huaier), which is a sandy beige mushroom from the trunk, has antitumor activity. Huaier could decrease the viabilities, numbers, and sizes of mammospheres. The clonogenicity of MCF7 breast cancer cell was impaired, along with less holoclones after Huaier exposure. Further, Huaier could decrease the proportion of cells expressing CD44^+^/CD24^-^, which exhibit cancer stem-like properties, and reduce the levels of stem cell markers including NANOG, NESTIN and OCT-4. Additionally, Huaier-mediated effect on CSCs was partially dependent on the hedgehog pathway (Wang et al., 2014). Another study showed that Huaier extract could inhibit the potential of spheroid formation and the population of aldehyde dehydrogenase (ALDH)−positive cell in primary CRC cells (T1 and T2 cells) by downregulating the Wnt/β−catenin pathway (Zhang et al., 2013). Taken together, these findings provide experimental evidence that Huaier extract is a promising TCM for suppressing CSCs in multiple cancers.

### TCM Could Induce Cell Death of Tumor Cells

#### Induction of Apoptosis

Induction of tumor cell death by apoptosis is a well-known anti-tumor strategy (Debatin, 2000; Xiu et al., 2015). Saponins are compounds of saponin and sugar, uronic acid or other organic acids, which are widely found in plants. The total saponins of *A. mongholicus* Bunge [Fabaceae] (AST) could induce the extrinsic apoptotic cascade and caused cell cycle arrest in HT-29 CRC cells by regulating both mTOR and ERK signaling pathways, of which inhibition of NF-kappaB is a critical latter event (Auyeung et al., 2010). *Laminaria japonica* is a traditional Oriental herbal medicine. Glycoprotein isolated from *L. japonica* (LJGP) exhibited anti-cancer activity in cultured human gastric carcinoma AGS cells. LJGP could inhibit tumor proliferation and induce apoptosis by upregulating pro-apoptotic Bax, and downregulating anti-apoptotic Bcl-2 and IAP family members, as well as activation of caspase-3/9. This was associated with downregulation of telomerase activity and prostaglandin E_2_ synthesis by decreasing the levels of cyclooxygenase (COX)-2 (Ho et al., 2011).

Fas/FasL-mediated signaling is critical in regulation of cell death. Fas binds to its ligand FasL with FADD (Fas-related death domain structure protein), to form the DISC (death-inducing signaling complex), which induces activation of caspase-8 and then cleaves effector caspases to induce apoptotic cell death (Zhang Y. S. et al., 2016). Physiologically, Fas^high^FasL^low^ cells combined with cytotoxic T cells (Fas^low^FasL^high^) expressed FasL, leading to the activation of Fas receptor and triggering apoptosis of target-cells (Villa-Morales and Fernandez-Piqueras, 2012). However, tumor cells with high-expressed FasL and low or nonfunctional Fas bound to lymphocytes expressed Fas and abrogated immune responses (Reichmann, 2002). A TCM formula PHY906 (*G. uralensis* Fisch. ex DC. [Fabaceae], *P. lactiflora *Pall. [Paeoniaceae], *S. baicalensis *Georgi [Lamiaceae], *Ziziphus jujuba* Mill. [Rhamnaceae]) could enhance the anti-tumor activity of Sorafenib in a nude mouse xenograft model of HepG2. *S. baicalensis* Georgi [Lamiaceae] (S), one herb of PHY906, was critical in increasing tumor apoptosis induced by Sorafenib with an increase of mouse FasL and human FasR expression (Lam et al., 2015). An herbal medicine formula Yang Wei Kang Liu (YWKLF) (*Panax ginseng* C.A.Mey. [Araliaceae], *Paris polyphylla* var. chinensis (Franch.) H. Hara [Melanthiaceae], *A. mongholicus* Bunge [Fabaceae], *Biancaea sappan* (L.) Tod. [Fabaceae], *S. diffusum *(Willd.) R.J.Wang [Rubiaceae], and *P. notoginseng* (Burkill) F.H.Chen [Araliaceae]) has been used for hundreds of years in China because of its antitumor activity. The sera from rabbits orally administered with YWKLF induced cell apoptosis in human gastric cancer MGC-803 cells through inducing mitochondrial dysfunction, increasing the expression of Fas and Bax, as well as reducing the mRNA of FasL (Li et al., 2008).

#### Induction of Autophagy

Autophagy is a “self-eating” process to maintain homeostasis that removes potentially injurious intracellular components and proteins. Induction of autophagy in tumor cells is promising to prevent tumor progression. There are physical and functional complex interactions and regulations between autophagy and programmed cell death. Although autophagy often accompanies programmed cell death following multiple toxic insults, the requirement of autophagic machinery for execution of programmed cell death is highly contextual (Denton and Kumar, 2019). Bufalin, the active compound of cinobufacini, is derivated from bufadienolide and developed in anaesthetic, cardiotonic and cancer treatment (Gao et al., 2011). Bufalin could inhibit the proliferation of human hepatoma cells including Huh7, Hep3B and HA22T and regulated the cell death program *in vitro*. Further, bufalin could induce autophagy in hepatoma cells by increasing TNF, MAPK and autophagy-related genes such as BECN-1 and ATG8, and decreasing Bcl-2 and Bid (Hsu et al., 2013). Dihydroartemisinin (DHA) and its analogs have anticancer activity. A novel DHA derivative, DHA-37 could induce cell death in multiple human cancer cell lines, such as lung carcinoma A549 cells and gastric cancer SGC-7901 cells. Mechanistically, DHA-37 could trigger autophagic cell death (ACD) in A549 cells by activation of the MAPK signal and upregulation of high mobility group protein (HMGB1). Additionally, in a murine xenograft model of A549, DHA-37 could inhibit tumor growth, and increase p-ERK, p-P38, HMGB1, and LC3 in tumor tissue, which is consistent with *in vitro* data (Liu et al., 2018). The root of *Platycodon grandiflorus* (Jacq.) A.DC. [Campanulaceae] (PG), a typical TCM herb, has anti-inflammatory (Zhang et al., 2015) and anti-tumor (Kim et al., 2005) activity. The platycoside-containing butanol fraction of PG (PGB) has been reported to induce ACD of A549 cells by suppressing the AKT/mTOR pathway and activating the AMPK and MAPK pathways (Ma et al., 2016). Flavonoids (FOJ) and steroidal saponins (SSOJ) are the main active components of *Ophiopogon japonicus* (Thunb.) Ker Gawl. [Asparagaceae] (OJ), which is used to adjust body functions and has anti-tumor activity (Zhang W. et al., 2016). FOJ or SSOJ could induce autophagy of A549 cells by upregulating protein levels of LC3-II and mRNA levels of Atg-3, Atg-7, Beclin-1 and LC3-II/I. Further study found that FOJ- or SSOJ-induced ACD was associated with inhibition of PI3K/Akt/mTOR signaling (Chen J. et al., 2017).

#### Induction of Necroptosis

Necroptosis is a form of programmed cell death; the canonical pathway of activation requires activation of mixed lineage kinase domain-like (MLKL) by receptor-interacting protein kinase 1 (RIP1)-RIP3 and further oligomerization and translocation of MLKL to the plasma membrane (Wu et al., 2020). Recent studies show that TCM also exerts anti-cancer effects through induction of necroptosis. Resibufogenin could suppress tumor growth and metastasis in a murine xenograft model of CRC. Further study showed that the anti-tumor effect of resibufogenin was associated with induction of necroptosis through increasing RIP3 and pMLKL (Han et al., 2018). Shikonin, one of the active ingredients of *Arnebia euchroma* (Royle ex Benth.) I.M.Johnst. [Boraginaceae], has anti-inflammatory, antioxidant, antimicrobial, antithrombotic, and antitumor effects (Andújar et al., 2013). Shikonin could induce necroptosis in rat C6 and human U87 glioma cells, which was mediated by RIP1-dependent signaling and oxidative stress (Huang C. et al., 2013).

#### Induction of Multiple Cell Death Pathways

Underlying mechanisms for the balance between programmed cell death including apoptosis and necroptosis, and autophagy are complex and contextual. With its characteristics, TCM has the synergistic effect on multiple cell death pathways. For example, *S. barbata *D.Don [Lamiaceae] (SB) is another typical TCM herb from the dried Labiatae plant. In addition to ER stress-, intrinsic mitochondrial-, P38/SIRT1-regulated cell apoptosis through G2/M phase arrest and extrinsic Fas/FasL-mediated pathways, autophagy also was important in SB-induced cytotoxicity in CL1-5 lung cancer cells. *In vivo*, SB could suppress proliferation and angiogenesis, and increase apoptosis and autophagy in CL1-5 tumor-bearing mice (Chen C. C. et al., 2017). Furthermore, Hibiscus leaf polyphenolic (HLP) extract could induce cell apoptosis through increasing the caspases cleavages, Bcl-2, and Fas/FasL activation; it also could induce ACD by increasing ATG5, Beclin1, and LC3-II in A375 melanoma cells (Chiu et al., 2015). In addition, a novel strategy for improving the DC-based cancer vaccine was provided in cancer treatment. Shikonin could trigger RIP1- and RIP3-dependent necroptosis in mouse stage IV mammary carcinoma 4T1-luc2 cells that is accompanied by enhanced autophagy. Shikonin-induced autophagy directly contributed to DAMP upregulation. Chloroquine-mediated inhibition of autophagy enhanced the surface DAMP activity and resulted in DC activation. *In vivo* study found that co-treatment of Shikonin with chloroquine stimulated DC activation and the derived vaccine efficacy in a murine model of mammary carcinoma (Lin et al., 2018).

## Clinical Applications

Many clinical randomized controlled trials have demonstrated that TCM could be beneficial as part of adjuvant chemotherapy in cancer treatment. YWKLF combined with chemotherapy prolonged the survival time compared to chemotherapy alone in a clinical trial of 123 patients with late stage gastric cancer (Li et al., 2008). Jianpi Bushen (JPBS) is used to invigorate the spleen and tonify the kidney. In a meta-analysis of randomized controlled trials, 26 studies with 3098 individuals were included to determine the effect of JPBS combined with chemotherapy for gastric cancer. JPBS treatment with regular chemotherapy improved the efficiency of clinical curative effect and quality of life (QOL) and immune response, and reduced side effects compared to chemotherapy alone (Chen et al., 2018). In a randomized controlled trial, herbal injection (Cinobufacini) and herbal decoction (benefitting Qi and Yin and detoxication recipe, *A. mongholicus* Bunge [Fabaceae], *A. macrocephala* Koidz. [Asteraceae], *Glehnia littoralis* (A.Gray) F.Schmidt ex Miq. [Apiaceae], *Asparagus cochinchinensis* (Lour.) Merr. [Asparagaceae], *Ligustrum lucidum* W.T.Aiton/Oleaceae), *Selaginella doederleinii* Hieron. [Selaginellaceae], *Salvia chinensis* Benth. [Lamiaceae], *P. polyphylla* var. chinensis (Franch.) H.Hara [Melanthiaceae], *P. vulgaris* L. [Lamiaceae], and *Ostreae concha*) were used in a randomized controlled trial to explore TCM actions as maintenance therapy in 64 patients with advanced NSCLC. Results showed that TCM maintenance therapy improved QOL and had higher 1-year survival of patients compared to those in the chemotherapy group (Jiang et al., 2016). In another randomized controlled trial of 106 patients with advanced NSCLC, TCM maintenance therapy (TCM decoction) improved 3-month progression-free survival (PFS) and QOL including physical well-being, emotional well-being and functional well-being (Han Y. et al., 2016).

In addition to NSCLC, a randomized controlled trial was conducted in 68 patients with HCC. Xiaoaiping (XAP), a TCM extracted from the roots of *Marsdenia tenacissima* (Roxb.) Moon [Apocynaceae], possessed antitumor activity and had been widely used in China for cancer treatment (Yu et al., 2019). Results showed that compared with the control group, the XAP group had improved immediate therapeutic efficacy and QOL and prolonged the PFS in patients. Moreover, levels of CD3^+^, CD4^+^ and CD4^+^/CD8^+^ in the peripheral blood in the XAP group were higher than those in the control group (Huang Z. et al., 2013). Furthermore, a clinical trial was conducted to explore the cellular immune regulatory effect of Bushen Jianpi Recipe (BSJPR) (*R. glutinosa* (Gaertn.) DC. [Orobanchaceae], *Cornus officinalis* Siebold & Zucc. [Cornaceae], *Dioscorea oppositifolia* L. [Dioscoreaceae], *C. pilosula* (Franch.) Nannf. [Campanulaceae], *A. macrocephala* Koidz. [Asteraceae], *S. glabra* Roxb. [Smilacaceae], *Alisma plantago-aquatica* L. [Alismataceae], *Paeonia × suffruticosa* Andrews [Paeoniaceae], *G. glabra* L. [Fabaceae]) in 117 patients with primary liver cancer after transcatheter arterial chemoembolization (TACE). BSJPR treatment with TACE improved TCM syndrome, half-year survival rate and QOL in patients compared to the control group. Laboratory examination showed combination therapy of BSJPR with TACE increased MHC class II (CD14^+^/HLA-DR), IFN-γ and IL-12 in monocytes (Wang et al., 2008). Additionally, Yiqi Jiedu Quyu Recipe (YJQR) contains (*A. mongholicus* Bunge [Fabaceae], *Pseudostellaria heterophylla* (Miq.) Pax [Caryophyllaceae], *S. diffusum* (Willd.) R.J.Wang [Rubiaceae], *Ranunculus ternatus* Thunb. [Ranunculaceae], *P. vulgaris* L. [Lamiaceae], *C. aromatica* Salisb. [Zingiberaceae], *C. longa* L. [Zingiberaceae], *Plantago asiatica* L. [Plantaginaceae], *Phellodendron amurense* Rupr. [Rutaceae], *Pyrrosia lingua* (Thunb.) Farw. [Polypodiaceae], *Foreknowledge*) In a randomized controlled trial of 44 patients with advanced prostate cancer (APC), YJQR treatment with endocrine therapy for 6 months increased prostate specific antigen (PSA), free PSA (f-PSA) and hemoglobin (Hb) in serum. Meanwhile, they also improved QOL and immune function as well as reduced adverse reactions (Jia Y.J et al., 2013). Taken together, these findings suggest that TCM has the potential in either combination therapy with regular chemotherapy or maintenance therapy for cancer (**Table 6**). However, large sample randomized controlled trials and additional rigorously designed are further required to confirm the efficacy and safety for TCM applications in patients.

**Table 6:** TCM in clinical applications.

TCM	Patients	Effects	Main mechanisms	Ref.
YWKLF (formula, orally) combined with chemotherapy	Stage IV gastric cancer, 123 patients	Prolonged the survival time compared to chemotherapy alone	The sera from rabbits orally administered with YWKLF induced cell apoptosis in human gastric cancer MGC-803 cells through inducing mitochondrial dysfunction, increasing the expression of Fas and Bax, as well as reducing the mRNA of FasL	(Li et al., 2008)
JPBS (formula, orally) combined with chemotherapy	Gastric cancer, 26 studies with 3098 individuals	Improved the efficiency of clinical curative effect and QOL of patients	Increase the levels of CD3^+^, CD4^+^, CD4^+^/CD8^+^, NK^+^, and macrophages in patient serum	(Chen et al., 2018)
Cinobufacini (TCM monomer, injection) and herbal decoction (formula, orally) maintenance therapy	NSCLC, 64 patients	Improved QOL and had higher 1-year survival of patients	Not mentioned	(Jiang et al., 2016)
TCM decoction (formula, orally), maintenance therapy	NSCLC, 106 patients	Improved 3-month PFS and QOL in patients	Not mentioned	(Han Y. et al., 2016)
XAP (TCM component, injection)	HCC, 68 patients	Improved immediate therapeutic efficacy and QOL and prolonged the PFS in patients	Increasing levels of CD3^+^, CD4^+^and CD4^+^/CD8^+^ in the peripheral blood	(Huang Z. et al., 2013)
BSJPR (formula) combined with TACE	primary liver cancer, 117 patients	Improved the half-year survival rate and QOL of patients	Increasing MHC class II (CD14+/HLA-DR), IFN-γ and IL-12 in monocytes	(Wang et al., 2008; Song et al., 2013)
YJQR (formula, orally) combined with endocrine therapy	APC, 44 patients	Increased PSA, f-PSA and Hb in serum, and improved QOL and immune function of patients	Increasing levels of CD3^+^, CD4^+^ and CD4^+^/CD8^+^ and NK cell in serum	(Jia Y. J. et al., 2013)

YWKLF, Yang Wei Kang Liu formula; JPBS, Jianpi Bushen; QOL, quality of life; herbal decoction, benefiting Qi and Yin and detoxication recipe; NSCLC, non-small cell lung cancer; PFS, progression-free survival; XAP, Xiaoaiping; HCC, hepatocellular carcinoma; BSJPR, Bushen Jianpi Recipe; TACE, transcatheter arterial chemoembolization; YJQR, Yiqi Jiedu Quyu Recipe; APC, advanced prostate cancer; PSA, prostate specific antigen; f-PSA, free PSA; Hb, hemoglobin.

## Conclusions and Challenges

Cancer remains a major threat to human life, which results in a huge healthcare burden. Most of therapeutic strategies targeting tumor cells ignore the importance of TIM. TIM plays a critical role in tumor development (Swartz et al., 2012). TIM includes abundant nontumor cell, stromal cells, extracellular matrix, cellular factors and chemokines, all of these promote tumor growth, invasion, and metastases, and protect tumor cells to escape from host immunity (Swartz et al., 2012). Therefore, understanding tumor cells and their interaction with TIM as a whole system is promising to have a better therapeutic effect.

**Figure 1:**
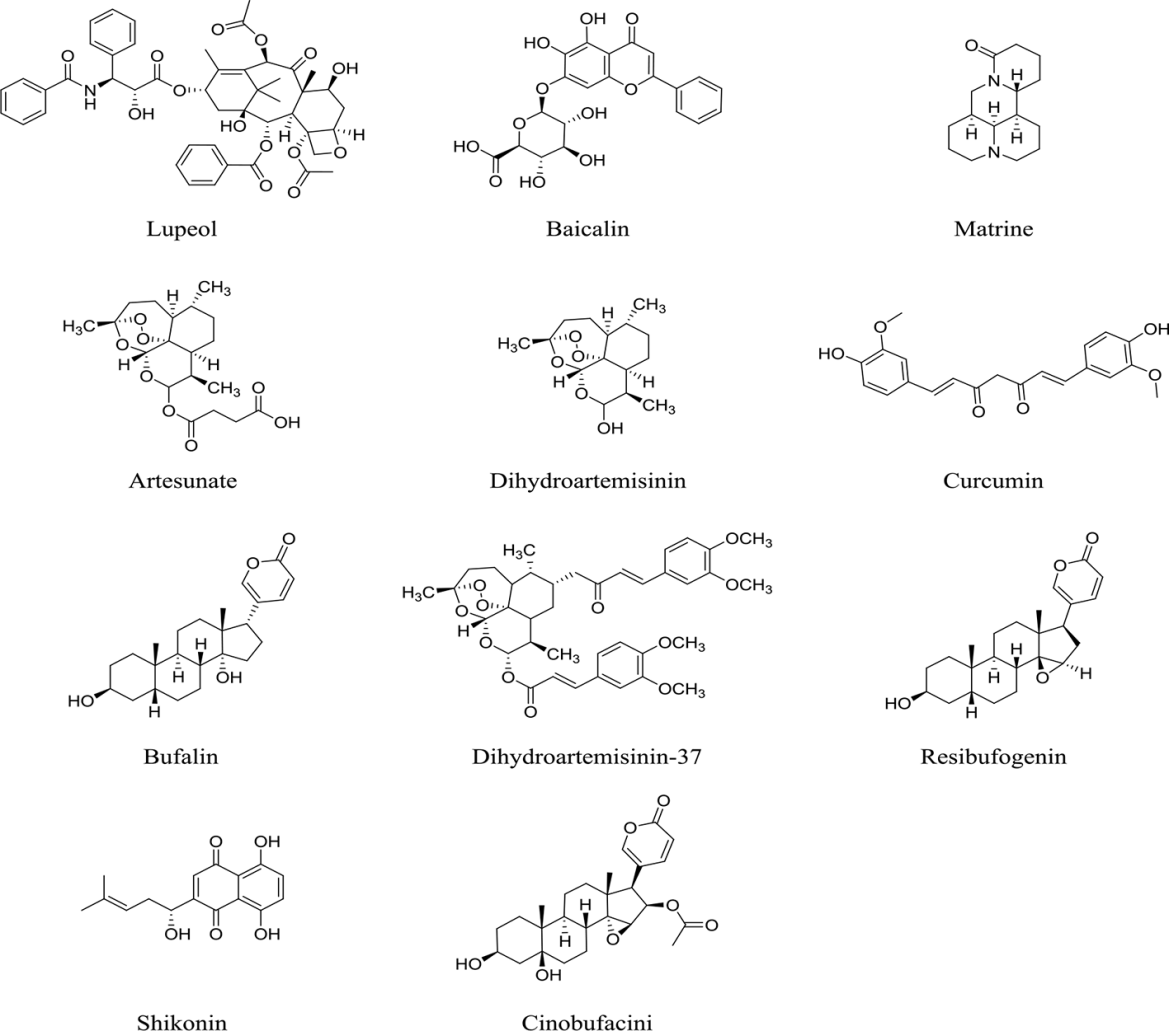
Chemical structures of multiple TCM monomers.

**Figure 2:**
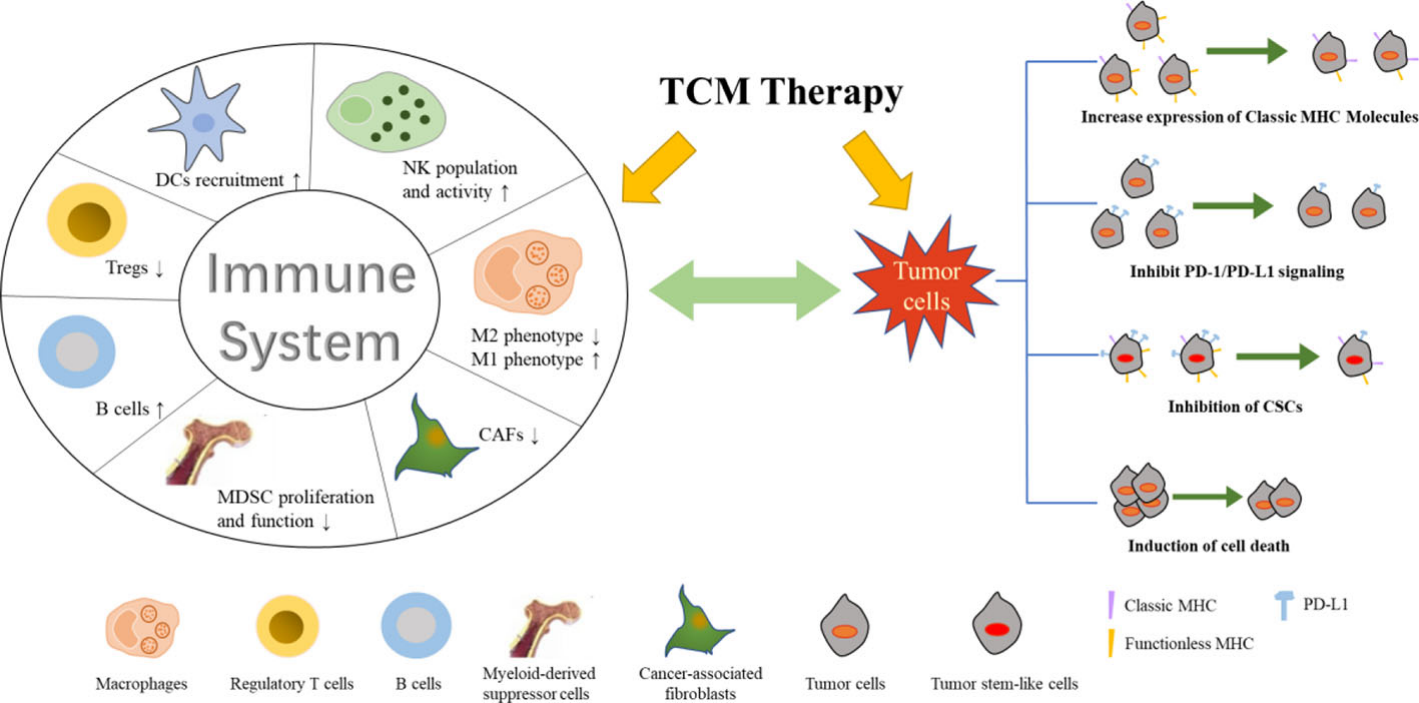
Underlying mechanism for effect of TCM on tumor cells and TIM.

Although the chemical composition in TCM formula is complex, this characteristic of TCM fits well with the treatment of TIM and tumor cells as a whole system. In contrast, western medicine has specific targets and strong effect; however, the toxic and side effects are also dramatic. such as dermatologic events associated with cixutumumab (Daher et al., 2011), severe cytotoxic with decitabine (Berdasco and Esteller, 2019). Levamisole hydrochloride was withdrawn due to its severe agranulocytosis. Most of the drugs can only slow disease progression, they are unable to achieve the goal of cure. In addition, the cost of developing anticancer drugs is huge, which makes it much harder to develop new drugs. Compared to western medicine, TCM has a milder effect and act synergistically on tumor-related multi-target groups. With the help of network pharmacology, TCM can better act on tumors and TIM and have a better anti-cancer effect. In conclusion, we summarized the underlying mechanisms by which TCM, including formula, medicinal herb and monomer (**Figure 1**), inhibits tumor cells and TIM, indicating that TCM suppresses tumor cells *per se*, as well as affects immune responses in TIM (**Figure 2**). More information regarding preparation of TCM and study conditions are shown in **Supplemental tables**. Additionally, TCM can enhance the therapeutic effect of radiotherapy and chemotherapy and reduce its toxicity, prolong lifetime. Therefore, in some cases, TCM may be an ideal treatment strategy for cancer due to its synergistic effect on tumor cells and TIM. However, the specific mechanism for effects of many TCM formulas on cancer still needs further to be investigated due to the chemical composition of TCM is very complicated.

Although TCM has good synergistic effects on TIM and cancer cells, many challenges remain. First, as we mentioned above, tumor cells interacted with TIM, however, the specific mechanism of the crosstalk between tumor cells and TIM is still not clear. Second, due to the complexity of TCM prescription, studies based on experiences limit the logic and repeatability, which remains an obstacle to draw certain conclusions. Third, safety of TCM is hard to guarantee because of lacking standardized and systemic operating procedures in processing TCM. Finally, clinical researches lack multi-center, randomized control, efficacy comparison, and large-samples. Therefore, an appropriate system, such as standardizing *in vitro* and *in vivo* models and complete quality control, should be developed to investigate TCM actions on multi-level and multi-channel.

## Author Contributions

Study concept and design: XW, ZL, YC. Researching papers: HH, XH, LihZ, LiaZ. Drafting of the manuscript: HH, XW. Critical revision of the manuscript for important intellectual content: XW, JF, XF, TM. Obtained funding: ZL, ZW, YC, XW.

## Funding

This work was supported by the National Key Research and Development Project (No. 2019YFC1708900), National Major Scientific and Technological Special Project for Significant New Drugs Development (2019ZX09204-001), National Natural Science Foundation of China (No.21772005), Natural Science Foundation of Anhui Province (1908085QH312) and Beijing Natural Science Foundation (7202088, 7172118).

## Conflict of Interest

The authors declare that the research was conducted in the absence of any commercial or financial relationships that could be construed as a potential conflict of interest.
