# Supplementary material for: Advances in Molecular Mechanisms for Traditional Chinese Medicine Actions in Regulating Tumor Immune Responses
Source: Front Pharmacol. 2020 Jul 8;11:1009. doi: 10.3389/fphar.2020.01009 (PMC7360845; doi:10.3389/fphar.2020.01009)
Supplement: Supplementary file 1 [file DataSheet_1.docx]

**Table S1**. Preparation of TCM formula.

| **TCM formula** | **Modes of preparation** | **Ref.** |
| --- | --- | --- |
| SLE | Extracted the mixture of *Sophorae Flos* (*Styphnolobium japonicum* (L.) Schott/Fabaceae) and *Lonicerae Japonicae Caulis* (*Lonicera japonica* Thunb./Caprifoliaceae) (5:1) with 30% (v/v) ethanol aqueous, and then lyophilized to give powdered SLE | (Liu et al., 2019) |
| YPF | Yu-Ping-Feng (YPF) granules were purchased commercially, containing *Astragali Radix* (*Astragalus mongholicus* Bunge/Fabaceae), *Atractylodis Macrocephalae Rhizoma* (*Atractylodes macrocephala* Koidz./Asteraceae), and *Saposhnikoviae Radix* (*Saposhnikovia divaricata* (Turcz. ex Ledeb.) Schischk./Apiaceae) (2: 2: 1) | (Luo et al., 2016) |
| BFD | Bu Fei Decoction (BFD) granules were purchased commercially, containing *Codonopsis Radix* (*Codonopsis pilosula* (Franch.) Nannf. /Campanulaceae), *Schisandrae Chinensis Fructus* (*Schisandra chinensis* (Turcz.) Baill./ Schisandraceae), *Rehmanniae Radix* (*Rehmannia glutinosa* (Gaertn.) DC./ Orobanchaceae), *Astragali Radix* (*Astragalus mongholicus* Bunge/Fabaceae), *Asteris Radix Et Rhizoma* (*Aster tataricus* L.f./Asteraceae), and *Mori Cortex* (*Morus alb*a L./Moraceae) (3:2:1.8:4:2:2), BFD granules were grinded into powder, dissolved in water and configured to a concentration of 1 g/ml crude drug, and then filtered to give mother liquor for experiment | (Pang et al., 2017) |
| SYY | Extracted the mixture of *Salviae Miltiorrhizae Radix Et Rhizoma* (*Salvia miltiorrhiza* Bunge/Lamiaceae), *Astragali radix* (*Astragalus mongholicus* Bunge/Fabaceae), *Lycii Cortex* (*Lycium barbarum* L./Solanaceae), *Crataegi Fructus* (*Crataegus pinnatifida* Bunge/Rosaceae), and *Trionyx sinensis* Wiegmann (3:3:5:5:5) with water, and then filtrated, concentrated, and soaked with ethanol to give Songyou Yin (SYY) | (Zhang et al., 2016a) |
| ISD | Invigorating Spleen and Detoxification Decoction (ISD) was purchased commercially, containing *Codonopsis Radix* (*Codonopsis pilosula* (Franch.) Nannf. /Campanulaceae), *Smilacis Glabrae Rhizoma* (*Smilax glabra* Roxb./Smilacaceae), *Atractylodis Macrocephalae Rhizoma* (*Atractylodes macrocephala* Koidz./Asteraceae), *Glycyrrhizae Radix Et Rhizomapraeparata Cum Melle* (*Glycyrrhiza uralensis* Fisch. ex DC./Fabaceae), *Bupleuri Radix* (*Bupleurum chinense* DC./Apiaceae), *Curcumae Rhizoma* (*Curcuma longa* L./Zingiberaceae), *Scutellariae Barbatae Herba* (*Scutellaria barbata* D.Don/Lamiaceae) (formulated in a predetermined ratio), extracted ISD with water to make a crude drug with concentration of 2 g/ml | (Li et al., 2014) |
| QYSL | The mixture of *Astragali Radix* (*Astragalus mongholicus* Bunge/Fabaceae) (30 g), *Polygonati Odorati Rhizoma* (*Polygonatum odoratum* (Mill.) Druce/Asparagaceae) (10 g), *Scolopendra* (6 g), *Pheretima* (6 g), *Solanum nigrum* L./Solanaceae (20 g), *Scleromitrion diffusum* (Willd.) R.J.Wang/Rubiaceae (20 g), *Coicis Semen* (*Coix lacryma-jobi* L./Poaceae) (20 g), *Euphorbia helioscopia* L./Euphorbiaceae (6 g), *Curcumae Rhizoma* (*Curcuma longa* L./Zingiberaceae) (10 g), *Fritillariae Cirrhosae Bulbus* (*Fritillaria cirrhosa* D.Don/Liliaceae) (6g) is extracted with water, then concentrate to give a crude drug with concentration of 4.024 g/mL | (Zhang et al., 2016b) |
| GQD | Gegen Qinlian decoction (GQD) was purchased commercially, containing *Puerariae Lobatae Radix* (*Pueraria montana var. lobata* (Willd.) Maesen & S.M.Almeida ex Sanjappa & Predeep/Fabaceae), *Scutellariae Radix* (*Scutellaria baicalensis* Georgi/Lamiaceae), *Coptidis Rhizoma* (Coptis chinensis Franch./Ranunculaceae), *Glycyrrhizae Radix Et Rhizomapraeparata Cum Melle* (*Glycyrrhiza uralensis* Fisch. ex DC./Fabaceae) (5:3:3:2). GQD is extracted with ethanol, then filter, the filtrate is evaporated to dryness under reduced pressure, and lyophilized to obtain powder | (Lv et al., 2019) |
| PZH | Pien Tze Huang (PZH) was purchased commercially, containing *Abelmoschus moschatus* Medik./Malvaceae, *Calculus Bovis*, Snake Gall and *Panax notoginseng* (Burkill) F.H.Chen*.*/Araliaceae. PZH powder is dissolved in phosphate-buffered saline (PBS) to a concentration of 40 mg/ml as mother liquor for experiment | (Wei et al., 2014) |
| PHY906 | Containing *Glycyrrhizae Radix Et Rhizomapraeparata Cum Melle* (*Glycyrrhiza uralensis* Fisch. ex DC./Fabaceae), *Paeoniae Radix Alba* (*Paeonia lactiflora* Pall./Paeoniaceae), *Scutellariae Radix (Scutellaria baicalensis Georgi/Lamiaceae)*, *Jujubae Fructus* (*Ziziphus jujuba* Mill./Rhamnaceae) | (Lam et al., 2015) |
| YWKLF | The mixture of *Astragali Radix* (*Astragalus mongholicus* Bunge/Fabaceae) (450 g), *Notoginseng Radix Et Rhizoma* (*Panax notoginseng* (Burkill) F.H.Chen/Araliaceae) (40 g), *Ginseng Radix Et Rhizoma* (*Panax ginseng* C.A.Mey./Araliaceae) (100 g), *Paridis Rhizoma* (*Paris polyphylla* var. chinensis (Franch.) H.Hara/ Melanthiaceae) (150 g), *Sappan Lignum* (*Biancaea sappan* (L.) Tod./Fabaceae) (60 g), *Scleromitrion diffusum (Willd.) R.J.Wang*/Rubiaceae (180 g) was homogenized and soaked in water for 24 h, then heated at 100 degrees for 1 h, filtered, the filtrate was concentrated and lyophilized to obtain YWKLF powder | (Li et al., 2008) |
| Benefiting Qi and Yin and detoxication recipe | The mixture of *Astragali Radix* (*Astragalus mongholicus* Bunge/Fabaceae) (15 g), *Atractylodis Macrocephalae Rhizoma* (*Atractylodes macrocephala* Koidz./Asteraceae) (9 g), *Glehniae Radix* (*Glehnia littoralis* (A.Gray) F.Schmidt ex Miq./Apiaceae) (15 g), *Asparagi Radix* (*Asparagus cochinchinensis* (Lour.) Merr./Asparagaceae) (12 g), *Ligustri Lucidi Fructus* (*Ligustrum lucidum* W.T.Aiton/Oleaceae) (12 g), *Selaginella doederleinii* Hieron./Selaginellaceae (30 g), *Salvia chinensis* Benth./Lamiaceae (30 g), *Paridis Rhizoma* (*Paris polyphylla* var. chinensis (Franch.) H.Hara/Melanthiaceae) (30 g), *Prunellae Spica* (*Prunella vulgaris* L./Lamiaceae) (15 g), and *Ostreae Concha* (30g) was prepared the decoction the Good Manufacturing Practice (GMP) specific to Chinese herbal medicine | (Jiang et al., 2016) |
| BSJPR | Bushen Jianpi Recipe (BSJPR), the mixture of *Rehmanniae Radix Praeparata* (*Rehmannia glutinosa* (Gaertn.) DC./Orobanchaceae) (15 g), *Corni Fructus* (*Cornus officinalis* Siebold & Zucc./Cornaceae) (9 g), *Dioscoreae Rhizoma* (*Dioscorea oppositifolia* L./Dioscoreaceae) (9 g), *Codonopsis Radix* (*Codonopsis pilosula* (Franch.) Nannf. /Campanulaceae) (9 g), *Atractylodis Macrocephalae Rhizoma* (*Atractylodes macrocephala* Koidz./Asteraceae) (9 g), *Smilacis Glabrae Rhizoma* (*Smilax glabra* Roxb./Smilacaceae) (15 g), *Alismatis Rhizoma* (*Alisma plantago-aquatica subsp. oriental*e (Sam.) Sam./Alismataceae) (9 g), *Moutan Cortex* (*Paeonia × suffruticosa* Andrews/Paeoniaceae)(9 g), and *Glycyrrhizae Radix Et Rhizomapraeparata Cum Melle* (*Glycyrrhiza uralensis* Fisch. ex DC./ Fabaceae) (6 g) was decocted into a decoction for patients to take | (Wang et al., 2008) |
| YJQR | Yiqi Jiedu Quyu Recipe (YJQR), the mixture of *Astragali Radix* (*Astragalus mongholicus* Bunge/Fabaceae) (30 g), *Pseudostellariae Radix* (*Pseudostellaria heterophylla* (Miq.) Pax/Caryophyllaceae) (15 g), *Ranunculi Ternati Radix* (*Ranunculus ternatus* Thunb./Ranunculaceae) (15 g), *Scleromitrion diffusum* (Willd.) R.J.Wang/Rubiaceae (15 g), *Akebiae Fructus* (*Akebia trifoliata* (Thunb.) Koidz./Lardizabalaceae) (15 g), *Curcumae Radix* (*Curcuma aromatica* Salisb./Zingiberaceae) (10 g), *Curcumae Longae Rhizom*a (*Curcuma longa* L./Zingiberaceae) (10 g), *Plantaginis Herba* (*Plantago asiatica* L./Plantaginaceae) (15 g), *Phellodendri Chinensis Cortex* (*Phellodendron chinense* C.K.Schneid./Rutaceae) (15 g), and *Pyrrosiae Folium* (*Pyrrosia sheareri* (Baker) Ching/Polypodiaceae) (15 g) was decocted into a decoction for patients to take | (Jia et al., 2013) |

**Table S2**. Preparation of TCM components.

| **TCM components** | **Modes of preparation** | **Ref.** |
| --- | --- | --- |
| Am and/or Cp | Extracted the root of *Astragali Radix* (*Astragalus mongholicus* Bunge/Fabaceae) and *Codonopsis Radix* (*Codonopsis pilosula* (Franch.) Nannf. /Campanulaceae) with ddH_2_O (1:10; weight:volume), and then filtered, fractionated, extracted and separated by column chromatography to obtain ethanol-partitioned fraction (molecular mass of more than 10 kDa) which was lyophilized for use as experimental material. The two phytoextracts of *Astragali Radix* and *Codonopsis Radix* were designated as Am and Cp, respectively. A mixture of Am and Cp (1:1) termed as [Am+Cp] | (Chang et al., 2015) |
| GP | polysaccharides from *Glycyrrhizae Radix Et Rhizoma* (*Glycyrrhiza uralensis* Fisch. ex DC*.*/Fabaceae), purchased commercially | (He et al., 2011) |
| PP-1 | Extracted *Polygonati Odorati Rhizoma* (*Polygonatum odoratum* (Mill.) Druce/Asparagaceae) with water, and then filtered and precipitated the filtrate with ethanol to give the polysaccharide precipitate, named PP-1. PP-1 was re-dissolved in Dulbecco’s modified Eagle’s medium (DMEM) media at concentrations of 3mg/ml, which is crude drug for experiment | (Han et al., 2016a) |
| EPS | After 7 days Flask culture of G1, a cultivated strain of *Cordyceps sinensis*, was filtered to get culture supernatant, and then concentrated and precipitated with ethanol, the precipitate was washed and dried and re-dissolved in water, and centrifuged and collected the supernatant, after lyophilize the supernatant, chromatographic separation, and finally obtain EPS (exopolysaccharide) powder upon dialysis and lyophilization | (Song et al., 2013) |
| Huaier | Huaier extrat, purchased commercially, is dissolved in DMEM, and then filtered and configured the mother liquor with a concentration of 100mg/ml for experiment | (Zhang et al., 2013; Wang et al., 2014) |
| AST | *Astragali Radix* (*Astragalus mongholicus* Bunge/Fabaceae), purchased commercially, was refluxed in methanol for 1 h, then phase separate the residue to obtain total saponins (AST), lyophilize AST and redissolved in water to give a mother liquor with a concentration of 10mg/ml for experiment | (Auyeung et al., 2010) |
| LJGP | *Laminaria japonica*, purchased commercially, was soaked in water for 6 h, and then filtered. The filtrate was precipitated with ethanol, filtered again, and the filtrate was concentrated, ammonium sulfate was added to precipitate the glycoprotein, dialyze the glycoprotein, then centrifuge, and concentrate the supernatant to obtain LGJP (Glycoprotein isolated from *Laminaria japonica*), LGJP was dissolved in dimethyl sulfoxide as a stock solution at 10 mg/ml concentration | (Ho et al., 2011) |
| PGB | The root of *Platycodonis Radix* (*Platycodon grandiflorus* (Jacq.) A.DC./Campanulaceae), purchased commercially, was refluxed with methanol for 3 h, then filter, evaporate the filtrate to dryness, and then conduct fractionation (solvent system: ethylacetate and butanol). Each fraction was used in the experiment. The platycoside-containing butanol fraction was called PGB | (Ma et al., 2016) |
| FOJ and SSOJ | The powder of *Ophiopogonis Radix* (*Ophiopogon japonicus* (Thunb.) Ker Gawl./Asparagaceae) (OJ) was refluxed with ethanol, then filter, concentrate the filtrate and chromatograph it, then elute with different eluents. The 30% ethanol fraction was lyophilized to obtain FOJ powder; the powder of OJ was refluxed with ethanol, then filter, the filtrate is first extracted with petroleum ether and then with water saturated n-butanol. The extract of saturated n-butanol was chromatographed and eluted with different eluents. The 70% ethanol fraction was lyophilized to obtain SSOJ powder | (Chen et al., 2017b) |
| SB | *Scutellariae Barbatae Herba* (*Scutellaria barbata* D.Don/Lamiaceae) (SB), purchased commercially, was extracted with methanol, and then filtered and evaporated the filtrate to dryness as a crude drug for experiment | (Chen et al., 2017a) |
| HLP | The leaves of *Hibiscus sabdariffa* L./Malvaceae was extracted with methanol, then filter and evaporate the filtrate to dryness, the residue was re-dissolved in water, first extract with hexane to remove the pigment, then extract the aqueous phase with ethyl acetate, the residue was lyophilized to obtain powder HLP | (Chiu et al., 2015) |
| XAP | Xiaoaiping (XAP), purchased commercially, is a Chinese herbal injection. XAP was from the root of *Marsdeniae Tenacissimae Caulis* (*Marsdenia tenacissima* (Roxb.) Moon/Apocynaceae) | (Huang et al., 2013b; Yu et al., 2019) |

**Table S3**. Information of TCM monomer.

| **TCM monomers** | **Family/Species** | **Ref.** |
| --- | --- | --- |
| Lupeol | Purchased commercially, *Tamarindus indica L.* [Fabaceae] | (Wu et al., 2013) |
| PSG-1 | A polysaccharide isolated from *Ganoderma atrum*, named PSG-1, with a purity of >99.8% | (Yu et al., 2015) |
| Baicalin | Purchased commercially, the root of *Scutellariae Radix* (*Scutellaria baicalensis* Georgi/Lamiaceae) | (Tan et al., 2015) |
| Matrine | Purchased commercially, *Sophorae Flavescentis Radix* (*Sophora flavescens* Aiton/Fabaceae) | (Aghvami et al., 2018) |
| ARS and DHA | Artemisinin (ART) derivatives were purchased commercially. ART is chemical extract from *Artemisiae Annuae Herba* (*Artemisia annua* L./Asteraceae), artemether (ARM), artesunate (ARS) and dihydroartemisinin (DHA) were synthesized derivatives of ART | (Yao et al., 2018) |
| Curcumin | Purchased commercially, *Curcumae Longae Rhizom*a (*Curcuma longa* L./Zingiberaceae) | (Wang et al., 2017; Qu et al., 2018) |
| Bufalin | Purchased commercially, toad | (Hsu et al., 2013) |
| DHA-37 | Dihydroartemisinicn-37 (DHA-37) was synthesized derivative of artemisinin, which is a sesquiterpene lactone extracted from Chinese herb *Artemisiae Annuae Herba* (*Artemisia annua* L./Asteraceae) | (Liu et al., 2018) |
| Resibufogenin | Purchased commercially, a tovena lactone compound extracted from toad | (Han et al., 2018) |
| Shikonin | Purchased commercially, one of the active ingredients of *Arnebiae Radix* (*Arnebia euchroma (Royle ex Benth.) I.M.Johnst.*/Boraginaceae) | (Huang et al., 2013a) |
| Cinobufacini | Purchased commercially, toad | (Jiang et al., 2016) |

**Table S4**. Experimental details of TCM studies included.

| **TCM** | **Model/cell line** | **Dose range** | **Minimal active con.** | **Mode of administration** | **Controls used** | **Duration** | **Ref.** |
| --- | --- | --- | --- | --- | --- | --- | --- |
| SL | *In vivo*, a murine xenograft model of B16F10 melanoma | 0.6 g/kg, 1.2 g/kg,2.4 g/kg | 0.6 g/kg | I.g. once/day | NC: 0.5% carboxymethyl cellulose-Na (CMC-Na)  PC: Dacarbazine (DTIC, 50 mg/kg) | 14 days | (Liu et al., 2019) |
| [Am+Cp] | *In vivo*, a murine orthotopic mammary carcinoma resection model | NM | NM | Tail vein-injected at 1, 8, and 15 days post  tumor resection | NC: PBS  PC: DC+TCL+LPS | 51 days | (Chang et al., 2015) |
| YPF | *In vivo*, a LLC-xenografted murine model | 116 mg/mouse | NM | I.g. once/day | NC: PBS | 14 days before inoculation | (Luo et al., 2016) |
| Lupeol (monomer) | *In vitro*, NK cells and gastric cancer cell lines（BGC823, N87 and HGC27） | 0.1 to 200 μg/ml | 0.1 μg/ml | NM | NM | 24, 48, and 72 h | (Wu et al., 2013) |
| PSG-1 (monomer) | *In vitro*, mouse sarcoma cell line (s-180) | 20, 40, 80, and 160 μg/mL | 20 μg/ml | NM | NM | 48 h | (Yu et al., 2015) |
|  | *In vivo*, a murine xenograft model of sarcoma | 100 mg/kg | NM | I.g. once/day | NC: saline | 10 days |  |
| BFD | *In vitro*, NSCLC cell lines（H1975 and A549） | 0-80 mg/ml | NM | NM | NM | 24 h | (Pang et al., 2017) |
|  | *In vivo*, nude mice xenograft models of NSCLC | 30, 60 g/kg | 30 g/kg | I.g. twice/day | NM | 21 days |  |
| Baicalin (monomer) | *In vivo*, a murine orthotopic HCC implantation model | 50 mg/kg | NM | I.g. once/2 days | NC: PBS | 35 days | (Tan et al., 2015) |
| SYY | *In vivo*, a murine xenograft model of liver cancer | 4 g/kg | NM | I.g. once/day | NM | 42 days | (Zhang et al., 2016a) |
| GP | *In vivo*, a murine xenograft model of H22 hepatocarcinoma | 250 mg/kg | NM | subcutaneously injected into the napes and backs once/day | NC: PBS  PC: intraperitoneally injected with Cyclophosphamide (10 mg/kg•d, 200μL) for 7 days | 14 days | (He et al., 2011) |
| Matrine (monomer) | *In vitro*, human ALL B-lymphocytes | 1.68, 3.4, and 6.75 mg/ml | 1.68 mg/ml | NM | NM | 1 h | (Aghvami et al., 2018) |
| ARS and DHA | *In vitro*, L-929-CAFs | 50 μM | NM | NM | NM | 24 h | (Yao et al., 2018) |
|  | *In vivo*, a murine orthotopic breast cancer implantation model | 100 mg/kg | NM | I.g. once/day | NC: 0.5% CMC-Na  PC: i.p.injection paclitaxel (10 mg/kg) once every 2 day | 28 days |  |
| Curcumin (monomer) | *In vitro*, pancreatic cancer cell lines (Capan-1, panc-1) | 5, 10 μM | 5 μM | NM | NM | 24 h (Panc1) or 36 h (Capan1) | (Wang et al., 2017; Qu et al., 2018) |
|  | *In vivo*, a nude mouse xenograft model of pancreatic cancer | 10 μM | NM | injected in the tail vein once | NC: control-medium  Trail: CAF-CM (10 µm curcumin pretreated) | Mice were sacrificed 8 weeks after injection |  |
| PP-1 | In vitro, prostate-CAFs | 2, 10, 50, 250, and 1250 μg/ml | 250 μg/ml | NM | NC: starch polysaccharide PC: fluorouracil | 48 h | (Han et al., 2016a) |
| EPS | *In vitro*, dendritic cell sarcoma (DCS) cell line | 12.5, 25, 50, 100 μg/ml | 12.5 μg/ml | NM | PC: Lipopolysaccharide (LPS, 1 μg/ml) | 48 h | (Song et al., 2013) |
| ISD | *In vivo*, in spleen-deficient liver cancer rats | 18.8, 37.5 g /kg | 18.8 g/kg | I.g. once/day | PC: Thymopentin (5mg, intramuscular injection, 2 times a week) | 42 days | (Li et al., 2014) |
| QYSL | *In vivo*, a LLC-xenografted murine model | 20.12, 40.24, 80.48 g/kg | 20.12 g/kg | I.g. once/day | PC: cisplatin (0.4 ml cisplatin was injected intraperitoneally on days 1, 3, and 5) | 10 days | (Zhang et al., 2016b) |
| GQD | *In vivo*, a murine xenograft model of CT26 CRC | 0.3, 1.5, 7.5 mg/kg | 1.5 mg/kg | I.g. once/day | NC: 0.5% CMC-Na solution) | 32 days | (Lv et al., 2019) |
| PZH | *In vitro*, HT-29 CRC stem-like SP cells | 0.25, 0.5, 1 mg/ml | 0.25 mg/ml | NM | PC: verapamil | 24 h | (Wei et al., 2014) |
| Huaier | *In vitro*, MCF7 breast cancer cells | 0.5, 1, 2 mg/ml (mammospheres) | 0.5 mg/ml | NM | NM | 7 days | (Wang et al., 2014) |
|  |  | 2, 4, 8 mg/ml (clonogenicity and the expression of CD44^+^/CD24^-^) | 2 mg/ml | NM | NM | 24 h |  |
|  | *In vitro*, primary CRC cells (T1 and T2 cells) | 0.5, 1 mg/ml (the formation of spheroids) | 0.5 mg/ml | NM | NM | 14 days | (Zhang et al., 2013) |
|  |  | 0.25, 0.5 mg/ml (ALDH‑positive cells) | 0.25 mg/ml | NM | NM | 7 days |  |
| AST | *In vitro*, HT-29 CRC cells | 60 μg/ml | NM | NM | NM | 12, 24, 48 or 72 h | (Auyeung et al., 2010) |
| LJGP | *In vitro*, AGS gastric cancer cells | 5, 10, 15, 20, 25 μg/ml | 5 μg/ml | NM | NM | 72 h | (Ho et al., 2011) |
| PHY906 | *In vivo*, a nude mouse xenograft model of HepG2 | 500 mg/kg | NM | I.g. twice per day | PC:  Sorafenib (30 mg/kg) for 7 days | 4 days | (Lam et al., 2015) |
| YWKLF | *In vitro*, human gastric cancer MGC-803 cells | 1.63 g/ml | NM | NM | NC: saline  PC:  Cisplatin (2 μg/ml) | 24 h | (Li et al., 2008) |
| Bufalin | *In vitro,* human hepatoma cancer Huh7, Hep3B and HA22T cells | 0.016, 0.08, 0.4, 2, 10 μM (cell viability) | 0.016 μM | NM | NM | 72 h | (Hsu et al., 2013) |
|  |  | 0.4μM (cell cycle) | NM | NM | NM | 0, 8, 12 and 24 h |  |
| DHA-37 | *In vitro*, multiple human cancer cell lines including A549, SGC-7901 | 1, 5, 10, 50 μM | 1μM | NM | NM | 12, 24, 48, 72 h | (Liu et al., 2018) |
|  | *In vivo*, a murine xenograft model of A549 | 12.5, 25, 50 mg/kg | 12.5 mg/kg | I.p. injection once/day | NC: DMSO was dissolved in 200 μl corn oil  PC: 5-Fluorouracil (20 mg/kg) | 20 days |  |
| PGB | *In vitro*, human lung carcinoma A549 cells | 50, 100, 200 μg/ml | 50 μg/ml | NM | NM | 24 h | (Ma et al., 2016) |
| FOJ | *In vitro*, human lung carcinoma A549 cells | 0.2, 0.4, 0.8, 1.6, 3.1, 6.1, 12.2, 24.4, 48.8 mg/ml | 3.1 mg/ml | NM | PC: 3-MA (an autophagy inhibitor,5 mM) | 24 h | (Chen et al., 2017b) |
| SSOJ |  | 0.3, 0.5, 1, 2, 4, 7.9, 15.8, 31.6, 63.2 mg/ml | 4 mg/ml | NM |  |  |  |
| Resibufogenin | *In vivo*, a murine xenograft model of CRC | 5, 10 mg/kg | 5 mg/kg | I.p. injection once/day | PC: parthenolide | 21 days | (Han et al., 2018) |
| Shikonin | *In vitro*, rat C6 glioma cells | 1.2, 2.4, 3.6, 4.8, 6 μmol/L | 1.2 μmol/L | NM | NM | 3 h | (Huang et al., 2013a) |
|  | *In vitro*, human U87 glioma cells | 2, 4, 6, 8, 12 μmol/L | 2 μmol/L | NM |  |  |  |
| SB | *In vitro*, human lung cancer CL1-5 cells | 0.1, 0.2, 0.4, 0.5 mg/ml | 0.1 mg/ml | NM | NM | 24h | (Chen et al., 2017a) |
|  | *In vivo*, a murine xenograft model of lung cancer | 60 mg/kg | NM | I.p. injection six  times/week | NM | 15 days |  |
| HLP | *In vitro*, human malignant melanoma A375 cells | 10, 20, 50, 100, 250 μg/ml | 100 μg/ml | NM | NM | 24 h | (Chiu et al., 2015) |
| Shikonin | *In vitro*, mouse stage IV mammary carcinoma 4T1-luc2 cells | 0-64 μM (necroptosis） | 1 μM | NM | NM | 24 h | (Lin et al., 2018) |
|  |  | 0.5, 1, 2, 5 μM (autophagy) | 0.5 μM | NM | NM | 24 h |  |
|  | *In vivo*, a murine model of mammary carcinoma | 5μM | NM | I.p. injection once a week | NC: NM  PC: doxorubicin (2mg/kg) | 2 weeks |  |
| YWKLF  combined with chemotherapy | Stage IV gastric cancer, 123 patients | 12 g | NM | Orally twice/day | Control: chemotherapy | NM | (Li et al., 2008) |
| JPBS | Gastric cancer, 26 studies with 3098 individuals | NM | NM | Orally | NM | NM | (Chen et al., 2018) |
| Cinobufacini | 64 patients with advanced NSCLC | 20 ml | NM | Intravenous injection once/day | PC: chemotherapy | Day 1-day 10 | (Jiang et al., 2016) |
| Herbal decoction |  | 160 ml | NM | Orally twice/day |  | Day 1-day 21 |  |
| TCM decoctions | NSCLC, 106 patients | 200 ml | NM | Orally twice/day | NM | NM | (Han et al., 2016b) |
| XAP | HCC, 68 patients | 40 ml | NM | Intravenous drip once/day | NC: only best supportive treatment (BST) | 30 days, two weeks interval, another 30 days | (Huang et al., 2013b) |
| BSJPR | primary liver cancer, 117 patients | NM | NM | Orally twice/day | PC: silymarin (0.48g) and vitamin C (0.3g) three times/day | 12 weeks | (Wang et al., 2008) |
| YJQR | APC, 44 patients | 300 ml | NM | Orally once/day | NM | 6 months | (Jia et al., 2013) |

NM: No mentioned, NC: Negative control, PC: Positive control. I.g.: intragastrically, i.p.: intraperitoneal.

**References**

Aghvami, M., Ebrahimi, F., Zarei, M.H., Salimi, A., Pourahmad Jaktaji, R., and Pourahmad, J. (2018). Matrine Induction of ROS Mediated Apoptosis in Human ALL B-lymphocytes Via Mitochondrial Targeting. *Asian Pac J Cancer Prev* 19(2)**,** 555-560. doi: 10.22034/APJCP.2018.19.2.555.

Auyeung, K.K., Mok, N.L., Wong, C.M., Cho, C.H., and Ko, J.K. (2010). Astragalus saponins modulate mTOR and ERK signaling to promote apoptosis through the extrinsic pathway in HT-29 colon cancer cells. *International Journal of Molecular Medicine* 26(3)**,** 341.

Chang, W.T., Lai, T.H., Chyan, Y.J., Yin, S.Y., Chen, Y.H., Wei, W.C., et al. (2015). Specific medicinal plant polysaccharides effectively enhance the potency of a DC-based vaccine against mouse mammary tumor metastasis. *PLoS One* 10(3)**,** e0122374. doi: 10.1371/journal.pone.0122374.

Chen, C.C., Kao, C.P., Chiu, M.M., and Wang, S.H. (2017a). The anti-cancer effects and mechanisms of Scutellaria barbata D. Don on CL1-5 lung cancer cells. *Oncotarget* 8(65)**,** 109340-109357. doi: 10.18632/oncotarget.22677.

Chen, J., Yuan, J., Zhou, L., Zhu, M., Shi, Z., Song, J., et al. (2017b). Regulation of different components from Ophiopogon japonicus on autophagy in human lung adenocarcinoma A549Cells through PI3K/Akt/mTOR signaling pathway. *Biomed Pharmacother* 87**,** 118-126. doi: 10.1016/j.biopha.2016.12.093.

Chiu, C.T., Hsuan, S.W., Lin, H.H., Hsu, C.C., Chou, F.P., and Chen, J.H. (2015). Hibiscus sabdariffa Leaf Polyphenolic Extract Induces Human Melanoma Cell Death, Apoptosis, and Autophagy. *Journal of Food Science* 80(3)**,** H649-H658. doi: 10.1111/1750-3841.12790.

Han, Q.R., Ma, Y., Wang, H., Dai, Y., Chen, C.H., Liu, Y.W., et al. (2018). Resibufogenin suppresses colorectal cancer growth and metastasis through RIP3-mediated necroptosis. *Journal of Translational Medicine* 16. doi: ARTN 201

10.1186/s12967-018-1580-x.

Han, S.Y., Hu, M.H., Qi, G.Y., Ma, C.X., Wang, Y.Y., Ma, F.L., et al. (2016a). Polysaccharides from Polygonatum Inhibit the Proliferation of Prostate Cancer-Associated Fibroblasts. *Asian Pac J Cancer Prev* 17(8)**,** 3829-3833.

Han, Y., Wang, H., Xu, W., Cao, B., Han, L., Jia, L., et al. (2016b). Chinese herbal medicine as maintenance therapy for improving the quality of life for advanced non-small cell lung cancer patients. *Complementary Therapies in Medicine* 24**,** 81-89. doi: <https://doi.org/10.1016/j.ctim.2015.12.008>.

He, X., Li, X., Liu, B., Xu, L., Zhao, H., and Lu, A. (2011). Down-regulation of Treg cells and up-regulation of TH1/TH2 cytokine ratio were induced by polysaccharide from Radix Glycyrrhizae in H22 hepatocarcinoma bearing mice. *Molecules (Basel, Switzerland)* 16(10)**,** 8343-8352. doi: 10.3390/molecules16108343.

Ho, H.M., Gi Young, K., Sung-Kwon, M., Wun-Jae, K., Taek-Jeong, N., and Yung Hyun, C. (2011). Apoptosis induction by glycoprotein isolated from Laminaria japonica is associated with down-regulation of telomerase activity and prostaglandin E2 synthesis in AGS human gastric cancer cells. *International Journal of Oncology* 38(2)**,** 577-584.

Hsu, C.M., Tsai, Y., Wan, L., and Tsai, F.J. (2013). Bufalin induces G2/M phase arrest and triggers autophagy via the TNF, JNK, BECN-1 and ATG8 pathway in human hepatoma cells. *Int J Oncol* 43(1)**,** 338-348. doi: 10.3892/ijo.2013.1942.

Huang, C., Luo, Y., Zhao, J., Yang, F., Zhao, H., Fan, W., et al. (2013a). Shikonin Kills Glioma Cells through Necroptosis Mediated by RIP-1. *Plos One* 8.

Huang, Z., Wang, Y., Chen, J., Wang, R., and Chen, Q. (2013b). Effect of Xiaoaiping injection on advanced hepatocellular carcinoma in patients. *Journal of Traditional Chinese Medicine* 33(1)**,** 34-38.

Jia, Y.J., Li, X.J., Li, C., and Zhao, C. (2013). [Clinical efficacy analysis of treating advanced prostate cancer by yiqi jiedu quyu recipe combined endocrine therapy]. *Zhongguo Zhong Xi Yi Jie He Za Zhi* 33(4)**,** 448-451.

Jiang, Y., Liu, L.-S., Shen, L.-P., Han, Z.-F., Jian, H., Liu, J.-X., et al. (2016). Traditional Chinese Medicine treatment as maintenance therapy in advanced non-small-cell lung cancer: A randomized controlled trial. *Complementary Therapies in Medicine* 24**,** 55-62. doi: <https://doi.org/10.1016/j.ctim.2015.12.006>.

Lam, W., Jiang, Z., Guan, F., Huang, X., Hu, R., Wang, J., et al. (2015). PHY906(KD018), an adjuvant based on a 1800-year-old Chinese medicine, enhanced the anti-tumor activity of Sorafenib by changing the tumor microenvironment. *Sci Rep* 5**,** 9384. doi: 10.1038/srep09384.

Li, J., Sun, G.Z., Lin, H.S., Pei, Y.X., Qi, X., An, C., et al. (2008). The herb medicine formula "Yang Wei Kang Liu" improves the survival of late stage gastric cancer patients and induces the apoptosis of human gastric cancer cell line through Fas/Fas ligand and Bax/Bcl-2 pathways. *Int Immunopharmacol* 8(9)**,** 1196-1206. doi: 10.1016/j.intimp.2008.04.007.

Li, Y.L., Sun, B.G., Xiang, T., Chen, Z.X., and Zhang, S.J. (2014). [Effect of invigorating spleen and detoxification decoction on MHC I/MHC II in spleen-deficiency liver cancer rats survival]. *Zhong yao cai = Zhongyaocai = Journal of Chinese medicinal materials* 37(3)**,** 454-460.

Lin, S.Y., Hsieh, S.Y., Fan, Y.T., Wei, W.C., Hsiao, P.W., Tsai, D.H., et al. (2018). Necroptosis promotes autophagy-dependent upregulation of DAMP and results in immunosurveillance. *Autophagy* 14(5)**,** 778-795. doi: 10.1080/15548627.2017.1386359.

Liu, X.F., Wu, J.J., Fan, M.L., Shen, C., Dai, W.L., Bao, Y.N., et al. (2018). Novel dihydroartemisinin derivative DHA-37 induces autophagic cell death through upregulation of HMGB1 in A549 cells. *Cell Death & Disease* 9. doi: ARTN 1048

10.1038/s41419-018-1006-y.

Liu, Y.X., Bai, J.X., Li, T., Fu, X.Q., Guo, H., Zhu, P.L., et al. (2019). A TCM formula comprising Sophorae Flos and Lonicerae Japonicae Flos alters compositions of immune cells and molecules of the STAT3 pathway in melanoma microenvironment. *Pharmacol Res* 142**,** 115-126. doi: 10.1016/j.phrs.2019.02.020.

Luo, Y., Wu, J., Zhu, X., Gong, C., Yao, C., Ni, Z., et al. (2016). NK Cell-Dependent Growth Inhibition of Lewis Lung Cancer by Yu-Ping-Feng, an Ancient Chinese Herbal Formula. *Mediators Inflamm* 2016**,** 3541283. doi: 10.1155/2016/3541283.

Lv, J., Jia, Y., Li, J., Kuai, W., Li, Y., Guo, F., et al. (2019). Gegen Qinlian decoction enhances the effect of PD-1 blockade in colorectal cancer with microsatellite stability by remodelling the gut microbiota and the tumour microenvironment. *Cell Death Dis* 10(6)**,** 415. doi: 10.1038/s41419-019-1638-6.

Ma, Jin, Yeul, Liang, Chun, Yim, et al. (2016). A platycoside-rich fraction from the root of Platycodon grandiflorum enhances cell death in A549 human lung carcinoma cells via mainly AMPK/mTOR/AKT signal-mediated autophagy induction. *Journal of Ethnopharmacology An Interdisciplinary Journal Devoted to Bioscientific Research on Indigenous Drugs*.

Pang, L., Han, S., Jiao, Y., Jiang, S., He, X., and Li, P. (2017). Bu Fei Decoction attenuates the tumor associated macrophage stimulated proliferation, migration, invasion and immunosuppression of non-small cell lung cancer, partially via IL-10 and PD-L1 regulation. *Int J Oncol* 51(1)**,** 25-38. doi: 10.3892/ijo.2017.4014.

Qu, C., Wang, Q., Meng, Z., and Wang, P. (2018). Cancer-Associated Fibroblasts in Pancreatic Cancer: Should They Be Deleted or Reeducated? *Integrative cancer therapies* 17(4)**,** 1016-1019. doi: 10.1177/1534735418794884.

Song, D., He, Z., Wang, C., Yuan, F., Dong, P., and Zhang, W. (2013). Regulation of the exopolysaccharide from an anamorph of Cordyceps sinensis on dendritic cell sarcoma (DCS) cell line. *Eur J Nutr* 52(2)**,** 687-694. doi: 10.1007/s00394-012-0373-x.

Tan, H.Y., Wang, N., Man, K., Tsao, S.W., Che, C.M., and Feng, Y. (2015). Autophagy-induced RelB/p52 activation mediates tumour-associated macrophage repolarisation and suppression of hepatocellular carcinoma by natural compound baicalin. *Cell Death Dis* 6**,** e1942. doi: 10.1038/cddis.2015.271.

Wang, Q., Qu, C., Xie, F., Chen, L., Liu, L., Liang, X., et al. (2017). Curcumin suppresses epithelial-to-mesenchymal transition and metastasis of pancreatic cancer cells by inhibiting cancer-associated fibroblasts. *Am J Cancer Res* 7(1)**,** 125-133.

Wang, W.H., Zhou, R.Y., and Yan, Z.P. (2008). [Regulatory effect of bushen jianpi recipe on cellular immunity of patients with primary liver cancer after intervention therapy]. *Zhongguo Zhong Xi Yi Jie He Za Zhi* 28(7)**,** 583-587.

Wang, X., Zhang, N., Huo, Q., Sun, M., Dong, L., Zhang, Y., et al. (2014). Huaier aqueous extract inhibits stem-like characteristics of MCF7 breast cancer cells via inactivation of hedgehog pathway. *Tumour Biol* 35(11)**,** 10805-10813. doi: 10.1007/s13277-014-2390-2.

Wei, L., Chen, P., Chen, Y., Shen, A., Chen, H., Lin, W., et al. (2014). Pien Tze Huang suppresses the stem-like side population in colorectal cancer cells. *Mol Med Rep* 9(1)**,** 261-266. doi: 10.3892/mmr.2013.1760.

Wu, X.T., Liu, J.Q., Lu, X.T., Chen, F.X., Zhou, Z.H., Wang, T., et al. (2013). The enhanced effect of lupeol on the destruction of gastric cancer cells by NK cells. *Int Immunopharmacol* 16(2)**,** 332-340. doi: 10.1016/j.intimp.2013.04.017.

Yao, Y., Guo, Q., Cao, Y., Qiu, Y., Tan, R., Yu, Z., et al. (2018). Artemisinin derivatives inactivate cancer-associated fibroblasts through suppressing TGF-β signaling in breast cancer. *Journal of Experimental & Clinical Cancer Research*.

Yu, F., Li, Y., Zou, J., Jiang, L., Wang, C., Tang, Y., et al. (2019). The Chinese herb Xiaoaiping protects against breast cancer chemotherapy-induced alopecia and other side effects: a randomized controlled trial. *J Int Med Res* 47(6)**,** 2607-2614. doi: 10.1177/0300060519842781.

Yu, Q., Nie, S.P., Wang, J.Q., Huang, D.F., Li, W.J., and Xie, M.Y. (2015). Toll-like receptor 4 mediates the antitumor host response induced by Ganoderma atrum polysaccharide. *J Agric Food Chem* 63(2)**,** 517-525. doi: 10.1021/jf5041096.

Zhang, Q.B., Meng, X.T., Jia, Q.A., Bu, Y., Ren, Z.G., Zhang, B.H., et al. (2016a). Herbal Compound Songyou Yin and Moderate Swimming Suppress Growth and Metastasis of Liver Cancer by Enhancing Immune Function. *Integr Cancer Ther* 15(3)**,** 368-375. doi: 10.1177/1534735415622011.

Zhang, T., Wang, K., Zhang, J., Wang, X., Chen, Z., Ni, C., et al. (2013). Huaier aqueous extract inhibits colorectal cancer stem cell growth partially via downregulation of the Wnt/beta-catenin pathway. *Oncol Lett* 5(4)**,** 1171-1176. doi: 10.3892/ol.2013.1145.

Zhang, X., Tong, J., and Li, Z. (2016b). [Qiyusanlong decoction inhibits the level of PD-1/PD-L1 in mice bearing Lewis lung carcinoma]. *Xi Bao Yu Fen Zi Mian Yi Xue Za Zhi* 32(6)**,** 770-774.
